# QTL analysis of the developmental response to L-glutamate in Arabidopsis roots and its genotype-by-environment interactions

**DOI:** 10.1093/jxb/erx132

**Published:** 2017-04-26

**Authors:** Pia Walch-Liu, Rhonda C Meyer, Thomas Altmann, Brian G Forde

**Affiliations:** 1 Lancaster Environment Centre, Lancaster University, Lancaster, UK; 2 Leibniz Institute of Plant Genetics and Crop Plant Research, Department of Molecular Genetics, Gatersleben, Germany

**Keywords:** Environmental interactions, epistatic effects, glutamate, natural variation, nitrate, QTL mapping, root architecture, root growth, temperature sensitivity

## Abstract

Primary root growth in Arabidopsis and a number of other species has previously been shown to be remarkably sensitive to the presence of external glutamate, with glutamate signalling eliciting major changes in root architecture. Using two recombinant inbred lines from reciprocal crosses between Arabidopsis accessions C24 and Col-0, we have identified one large-effect quantitative trait locus (QTL), *GluS1*, and two minor QTLs, *GluS2* and *GluS3*, which together accounted for 41% of the phenotypic variance in glutamate sensitivity. The presence of the *GluS1* locus on chromosome 3 was confirmed using a set of C24/Col-0 isogenic lines. *GluS1* was mapped to an interval between genes At3g44830–At3g46880. When QTL mapping was repeated under a range of environmental conditions, including temperature, shading and nitrate supply, a strong genotype-by-environment interaction in the controls for the glutamate response was identified. Major differences in the loci controlling this trait were found under different environmental conditions. Here we present evidence for the existence of loci on chromosomes 1 and 5 epistatically controlling the response of the *GluS1* locus to variations in ambient temperature, between 20°C and 26°C. In addition, a locus on the long arm of chromosome 1 was found to play a major role in controlling the ability of external nitrate signals to antagonize the glutamate effect. We conclude that there are multiple loci controlling natural variation in glutamate sensitivity in Arabidopsis roots and that epistatic interactions play an important role in modulating glutamate sensitivity in response to changes in environmental conditions.

## Teaser

A major QTL controlling glutamate sensitivity of Arabidopsis root development has been mapped and additional loci identified that epistatically regulate its response to environmental changes.

## Introduction

Root growth and branching are highly responsive to a wide range of biotic and abiotic stimuli that play a major role in shaping the development of the root system. Amongst the most important abiotic factors affecting root development are the availability and spatial distribution of nutrients within the soil. The enhancement of root growth within fertile soil patches was reported 150 years ago (Nobbe, 1862) and a century later Wiersum (Wiersum, 1958) demonstrated that nitrate-rich zones of soil stimulated root branching. Drew (1975) reported that localized supplies of ammonium and phosphate, but not potassium, were also able to trigger a similar localised response as that elicited by localised nitrate. These responses are considered to be examples of foraging responses because of the way that they enable more precise placement of roots within those soil zones where the greatest benefit will be obtained (Hutchings and de Kroon, 1994). More recently, it was reported that different, but equally dramatic, changes in root architecture could be elicited when roots of a number of species, including Arabidopsis, are exposed to the amino acid L-glutamate (Glu) (Walch-Liu *et al.*, 2006). In Arabidopsis, the response to Glu consisted of a slowing or cessation of primary root (PR) growth, with apical meristem activity being the primary target, and a stimulation of lateral root outgrowth behind the PR tip. Lateral roots also became sensitive to Glu but the response was developmentally delayed until they were >5–10 mm long (Walch-Liu *et al.*, 2006).

Amino acids, including Glu, represent a significant proportion of the soluble N pool in unfertilized soils (Christou *et al.*, 2005). Roots are known to possess a set of high affinity amino acid transport systems capable of absorbing these amino acids from the rhizosphere at naturally occurring concentrations (Svennerstam *et al.*, 2011). Although the extent to which plants are able to utilise organic forms of soil N is still unclear (Jones *et al.*, 2005; Moran-Zuloaga *et al.*, 2015), it has been suggested that a localised source of Glu in the soil could act as a cue to trigger increased root branching within an organic N-rich patch, enhancing the plant’s ability to compete with microbes and other plants for uptake of the available amino acids within that patch (Forde and Lea, 2007). According to this hypothesis the root response to Glu represents another kind of foraging response, serving to enhance the precision of root placement within the soil with respect to heterogeneously distributed sources of organic N.

There are a number of lines of evidence that the root response to Glu involves a signalling effect, rather than a nutritional one. These include the finding that the effect is not elicited by glutamine or other related amino acids, the relatively low concentrations of Glu (50 µM) that are needed to trigger the response and the requirement for the PR tip to be in direct contact with the external Glu (Walch-Liu *et al.*, 2006; Forde, 2014). The case for a signalling role for Glu was further strengthened by the finding that MEKK1, a MAP kinase kinase kinase best known for its role in defence signalling, has a key role in eliciting the root’s response to Glu (Forde *et al.*, 2013).

A particularly striking aspect of the Glu response is the degree of natural variation in Glu sensitivity amongst Arabidopsis accessions, with C24 being the most sensitive of those tested and Col-0 being one of the least sensitive (Walch-Liu *et al.*, 2006). Natural variation has proved to be a powerful tool for the genetic analysis and dissection of complex traits in Arabidopsis (Weigel, 2012). Previous studies have found that combining naturally occurring genetic variation with multiple environmental treatments can increase the power of this approach (Tonsor *et al.*, 2005). This paper describes the results of a multi-environment study aimed at using natural variation in Arabidopsis to investigate the genetic control of the Glu response and its interactions with nitrate and other environmental factors. The results reveal a remarkable level of complexity in the genetic control of Glu sensitivity in Arabidopsis roots and in the genotype-by-environment interactions that modulate this sensitivity.

## Materials and methods

### Plant material

*Arabidopsis thaliana* L. (Heynh.) ecotypes were originally sourced from Lehle Seeds (Round Rock, TX, USA). The F_8_ recombinant inbred lines (RILs) were previously developed from reciprocal crosses between the Arabidopsis accessions Col-0 and C24 and genotyped using a set of 110 framework single nucleotide polymorphism (SNP) markers (Törjék *et al.*, 2006). Reciprocal sets of introgression (ILs) between Col-0 and C24 were generated and mapped as described previously (Törjék *et al.*, 2008).

### Plant growth and phenotyping

Seedlings were cultured vertically on 1% Phytagel plates containing a dilute basal medium at pH 5.7 (see supplementary Table S1 at *JXB* online) with 0.5% sucrose and 0.5 mM glutamine as the background N source (Walch-Liu and Forde, 2008). After sowing on 90 mm Petri dishes, sterilized seeds were stratified in the dark for 2 d at 4°C. The plates were then transferred to a growth room with a light intensity of ~100 µmol m^-2^ s^-1^ or a growth cabinet (Snijders, Tilburg, The Netherlands) with a light intensity of 330 µmol m^-2^ s^-1^, at the required temperature and for a 16/8 h photoperiod. After 4 d, seedlings were transferred either to 90 mm diameter Petri dishes, with 3 seedlings per plate, or 120 × 120 mm square plates, with 6 seedlings per plate, onto which either 50 µM K glutamate or 50 µM KCl had been added. When nitrate treatments were applied, KNO_3_ was added to a concentration of 5 mM and additional KCl was added to control treatments to maintain a uniform K^+^ concentration. After transfer, the positions of the PR tips were marked on the base of the plates and growth was continued under the same conditions. After a further 5 d, roots were imaged using a flatbed digital scanner and root growth analysed using Optimas Image Analysis software (Version 6.1, Media Cybernetics Inc., Silver Spring, MD, USA). In the multi-environment experiment, the shading treatment was achieved by suspending a steel plate containing an array of 5 mm square holes above the plates, reducing the light intensity reaching the seedlings from 330 µmol m^-2^ s^-1^ to 130 µmol m^-2^ s^-1^.

### QTL analysis

Composite Interval Mapping was performed using PLABQTL software (Utz and Melchinger, 1996) using the mean values of PBT and PAT_Glu_ for each RIL. Cofactors used for calculation were automatically chosen by the PLABQTL program by forward selection. Permutation analysis, using 1000 permutations, was performed to calculate the critical log of the odds (LOD) score (α = 0.05). Genotypic data used for the analysis were as previously determined (Törjék *et al.*, 2003).

### Fine mapping of ILs

Total DNA was extracted from leaves of selected ILs using the Qiagen Plant DNeasy minikit according to the manufacturer’s instructions. SNPs between Col-0 and C24 in the regions of interest were identified using the Arabidopsis GEBrowser (http://signal.salk.edu/atg1001/3.0/gebrowser.php) and dCAPS primers designed with the aid of dCAPS Finder 2.0 software (Neff *et al.*, 2002). MarkerTracker software (http://bbc.botany.utoronto.ca/markertracker/index.spy) was used to identify suitable PCR primers for CAPS (Cleaved Amplified Polymorphism) markers (Konieczny and Ausubel, 1993). PCR reactions were performed for 35 cycles using DreamTaq DNA polymerase (ThermoFisher Scientific) according to the manufacturer’s instructions. After digestion with the appropriate restriction enzyme, PCR reactions were electrophoresed on 1.5% agarose gels and stained with ethidium bromide to visualise the DNA fragments. Details of the SNPs, primers and restriction enzymes used in mapping are in Supplementary Table S2.

## Results

### Using RILs to map major QTLs controlling the Glu response

**Fig. 1.**
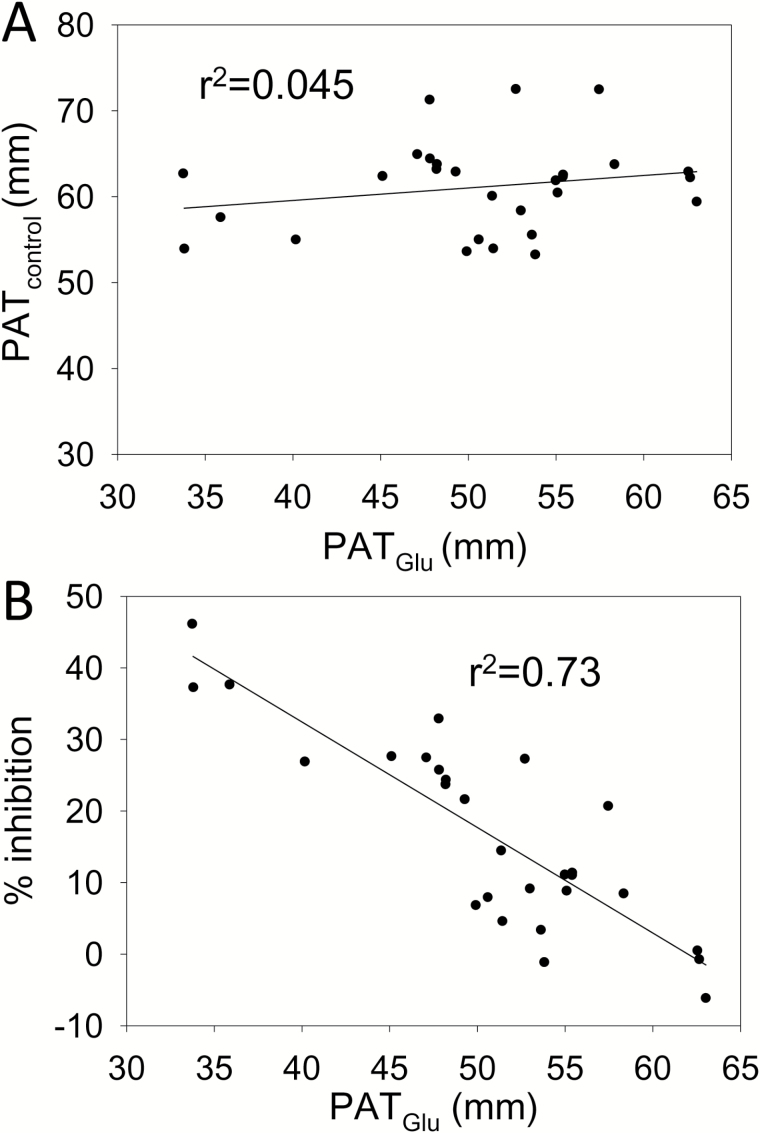
Correlation between different root growth parameters in the presence and absence of Glu for a set of 28 C24/Col-0 RILs. RILs were germinated and grown for 4 d before seedlings were transferred to nutrient agar plates 90 mm in diameter, with 3 seedlings per plate and 3 plates per treatment. The plates contained 50 µM K glutamate or 50 µM KCl (control). Growth was continued for 6 d. For each RIL the mean increase in length of PRs after transfer to Glu (PAT_Glu_), the mean increase in length of PRs after transfer to control plates (PAT_control_) and the percentage inhibition of PR elongation by Glu was determined. (A) PAT_Glu_*vs* PAT_control_. (B) PAT_Glu_ versus percentage inhibition. Linear regressions based on the least squares method were plotted using Sigmaplot (Ver. 8.0).

Strong differences previously observed between C24 and Col-0 in their sensitivity to low concentrations of Glu (Walch-Liu *et al.*, 2006) led us to choose a C24/Col-0 population of RILs (Törjek *et al.*, 2006) to map QTLs controlling the Glu response. A preliminary experiment was performed to establish the suitability of the C24/Col-0 RIL population for this analysis and to identify the most appropriate parameter for assaying the Glu response. A set of 28 RILs was grown on medium with and without 50 µM Glu, a concentration chosen because it was previously shown to strongly inhibit PR growth in C24 but to have almost no effect on Col-0 (Walch-Liu *et al.*, 2006). Amongst the RILs, a high degree of variation in the responsiveness to Glu was observed, values for % inhibition ranging from 0 to 46%, with a mean of 16.7% and a coefficient of variation of 0.80. When the increase in PR length in the 5 d after transfer to Glu (PAT_Glu_) was plotted for each RIL against PR growth in the control, no Glu, plates (Fig. 1A), there was only a weak correlation (r^2^ = 0.045). This showed that growth in the presence of Glu was largely determined by the root’s response to Glu and that intrinsic genetically determined differences in growth rate played little part. In agreement with this, when percentage inhibition was plotted against PAT_Glu_ (Fig. 1B), there was a strong negative correlation (r^2^ = 0.73). On this basis PAT_Glu_ rather than percentage inhibition was chosen as the parameter to use when analysing larger numbers of RILs for their Glu sensitivity, since it avoided the need for a control treatment for each line and therefore allowed more lines and/or treatments to be analysed in each experiment.

**Fig. 2.**
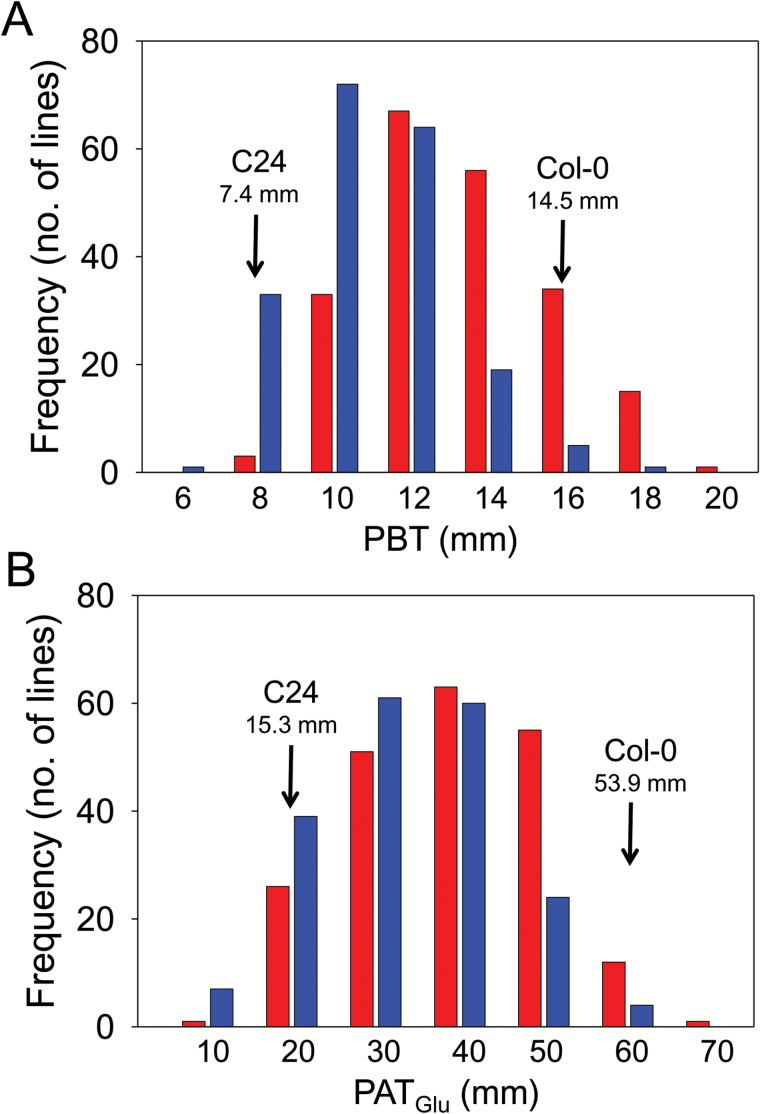
Frequency distribution plots of PR growth for a C24/Col-0 RIL population before and after transfer to Glu. A set of 368 RILs were germinated in a growth room at 21°C, with a low light intensity of 100 µEinsteins. Then 4 d-old seedlings were transferred to plates containing 50 µM K glutamate for the Glu sensitivity assay, with 3 seedlings per plate and 3 plates per line. (A) Frequency plot of PR length before transfer (PBT). (B) Frequency plot of PR growth after transfer to Glu (PAT_Glu_). Data for the two RIL populations have been plotted separately: Col-0 × C24 (red), C24 × Col-0 (blue). Mean values for the parental lines are indicated with arrows.

Using this approach, the root response to Glu was assayed using two populations of RILs, one comprising 193 lines derived from a Col-0 × C24 cross and the other comprising 175 lines derived from the reciprocal C24 × Col-0 cross (Törjek *et al.*, 2006). The growth of the PRs in the 4 d period before transfer (PBT) and their growth in the 5 d period after transfer to plates containing Glu (PAT_Glu_) were measured for each RIL and the frequency distributions for each trait are shown separately for the two populations in Fig. 2A and B. Both traits display a high degree of phenotypic variation with an essentially normal distribution, indicating the involvement of multiple genes, with only a small amount of transgressive segregation with respect to the parental lines. The ranked means of PBT and PAT_Glu_ for all lines used in this analysis, together with standard errors, have been plotted in Supplementary Figs S1 and S2, respectively. Since it was these mean values that were used for the QTL analysis, it is important to note the extent to which differences between the means are statistically significant across the two populations of RILs. The broad-sense heritabilities for the two traits were high in both RIL populations (*H*^*2*^ = 0.80 and 0.71 for PBT; *H*^*2*^ = 0.85 and 0.81 for PAT_Glu_), indicating their suitability for QTL mapping.

**Fig. 3.**
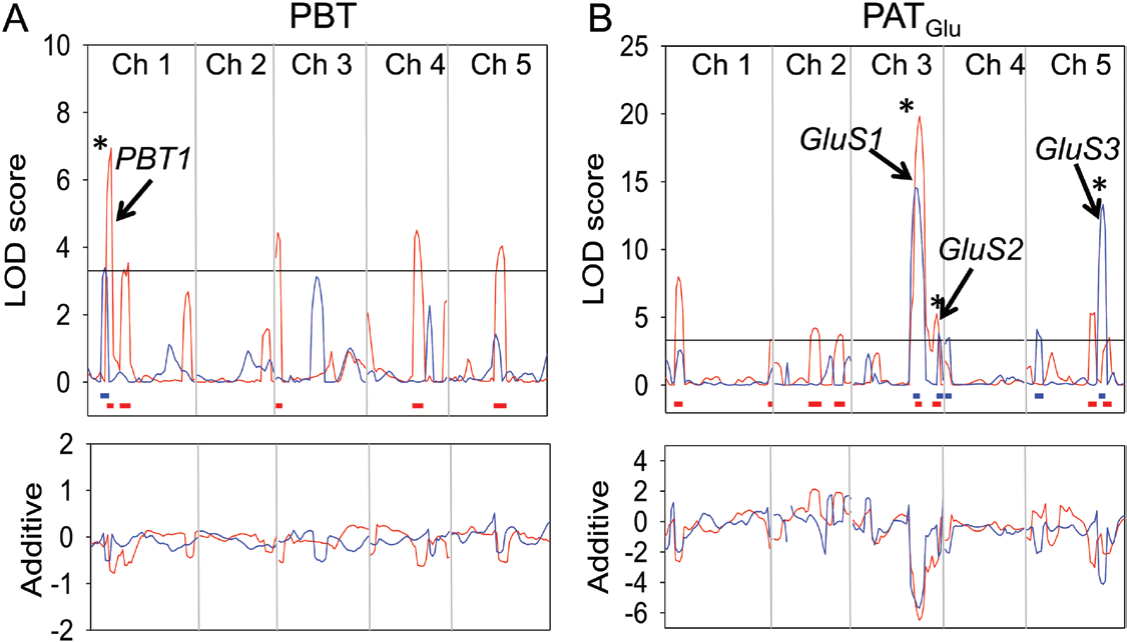
LOD score plots obtained by CIM analysis of PR growth traits in the C24/Col-0 RIL population. PR growth traits (PBT and PAT_Glu_) were determined for the Col-0 × C24 and C24 × Col-0 RIL populations as described in Fig. 2. QTL analysis was performed using PLABQTL software (Utz and Melchinger, 1996) and the LOD scores plotted separately for the Col-0 × C24 RIL population (red) and the C24 × Col-0 RIL population (blue) on the chromosome map. (A) PR growth before transfer (PBT); (B) PR growth after transfer to 50 µM Glu (PAT_Glu_). LOD significance thresholds (α = 0.05) were determined by 1000 permutations for each population, 3.3 for PBT and 3.2 for PAT_Glu_, and are shown as horizontal lines. The support intervals with a LOD fall-off of 1.0 for each significant QTL peak are shown as blue or red horizontal lines below the peaks. Asterisks indicate where significant QTL peaks in the two populations coincided. The lower graphs show plots of the additive effect of each region on the phenotype with respect to the C24 allele.

QTL analysis by composite interval mapping was performed using PLABQTL software. LOD score plots for the PBT and PAT_Glu_ traits are shown in Fig. 3. Three QTLs for PAT_Glu_ were identified whose LOD scores exceeded the threshold in both RIL populations (Fig. 3B). The strongest of these, *Glu Sensitivity 1* (*GluS1*), accounted for an average of 22.5% of the phenotypic variance for this trait in the two populations and mapped in the vicinity of MASC01171 on chromosome 3 with peak LOD scores at 58 and 62 cM, respectively. The other QTLs were located in the vicinity of MASC02788 on chromosome 3, *GluS2* with peak LOD scores at 78 and 80 cM, and MASC04394 on chromosome 5, *GluS3* with peak LOD scores at 72 and 78 cM. *GluS2* and *GluS3* accounted for an average of 7.5% and 12.3% of the phenotypic variance, respectively (see Table 1). None of these QTLs co-located with a significant peak in the PBT plots (Fig. 3A), supporting the conclusion that they are related to Glu sensitivity rather than to intrinsic variations in root growth rate. It was notable that for all three QTLs their allelic effect was negative with respect to the C24 allele, indicating that in each case it was the C24 allele that conferred an increased sensitivity to Glu. In this experiment only one QTL for the PBT trait, *PBT1*, was detected in both RIL populations (Fig. 3A and Supplementary Table S3). *PBT1* mapped to a region near the top of chromosome 1, with peak LOD scores at 16 and 22 cM.

**Table 1.** Summary of the QTLs controlling Glu sensitivity in the Col-0/C24 RIL population based on CIM mapping of PR growth in the presence of Glu (PAT_Glu_)

Environment		Exp’t	QTL ^ a ^	Peak ^ b ^	SI ^ c ^	Left marker ^ d ^	LOD	Allelic effect ^ e ^	pR ^ 2 ^ (%) ^ f ^	R ^ 2 ^ (%) ^ g ^	SD ^ g ^
Temperature (°C)	Other conditions										
21	Low light	Col-0x C24	*GluS1* *GluS2* *GluS3*	3/62 3/78 5/78	58–64 74–82 72–80	MASC01171 MASC03218 MASC04394	19.81 5.24 3.52	−6.138 −2.771 −1.532	30.76 10.78 3.16	66.5	3.9
		C24x Col-0	*GluS1* *GluS2* *GluS3*	3/58 3/80 5/72	56–62 78–84 68–74	MASC01171 MASC02788 MASC04591	14.54 3.84 13.31	−3.967 −1.927 −4.252	14.28 4.29 21.43	47.9	5.5
20	High light	Rep.1	*GluS1*	3/58	54–60	MASC01171	12.03	−4.214	32.05	43.8	7.9
		Rep.2	*GluS1*	3/56	54–62	MASC04819	6.18	−5.095	32.32	40.4	8.1
24	High light	Rep.1	*GluS1*	3/52	50–56	MASC05045	6.55	−3.917	8.41	43.9	7.9
		Rep.2	*GluS1*	3/58	54–62	MASC01171	4.84	−6.770	18.63	18.6	7.5
26	High light	Rep.1	*GluS4*	5/14	12–18	MASC05127	7.95	−7.331	22.37	22.4	7.8
		Rep.2	*GluS4*	5/14	10–18	MASC05217	4.76	−6.815	16.03	29.5	8.2
24	High light	Rep.1	*GluS5*	4/6	2–8	MASC04725	6.15	−5.935	22.67	44.0	7.9
	+nitrate	Rep.2	*GluS5*	4/2	0–6	MASC04123	4.21	−4.866	14.67	28.4	8.1

^a^Only those QTLs whose LOD score exceeded the threshold in the populations from both reciprocal crosses, or in both of the replicate experiments that were performed at each environmental condition in the multi-environment experiment, are shown. (Note that no reproducible QTLs were detected in the 24°C + shade treatment).

^b^Position of the highest LOD score in the QTL region (as chromosome/cM).

^c^The support interval (SI) within a LOD decrease of 1.0 from the QTL peak.

^d^The physical and genetical positions of the MASC framework markers are listed in Supplementary Table S5.

^e^Contribution of individual QTLs to the phenotypic variation.

^f^Effect of carrying the C24 allele at the respective position.

^g^R^2^ and SD give the explained phenotypic variation and associated standard deviation obtained from the final simultaneous fit of all putative QTLs in PLABQTL.

### Temperature sensitivity of the Glu response and its genotype dependence

**Fig. 4.**
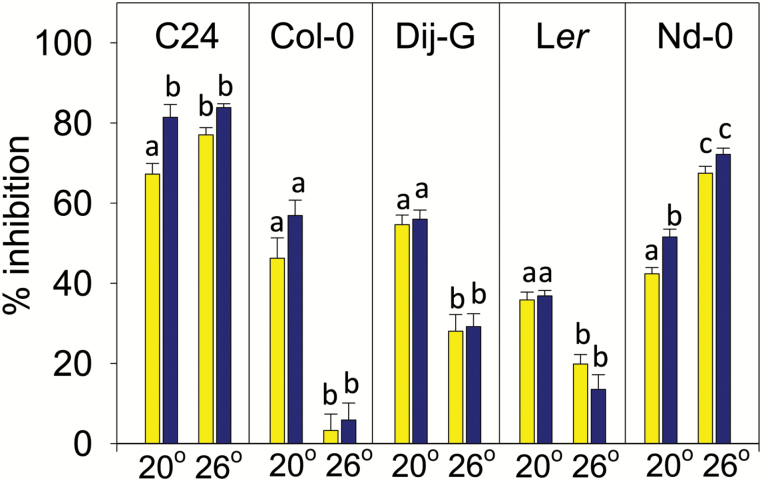
Effect of temperature on the Glu sensitivity of PR growth in five Arabidopsis accessions. Seedlings, which were 4 d-old, of each accession were transferred to treatment plates with and without Glu and grown under a light intensity of 250 µEinsteins for a further 6 d in separate growth cabinets at either 20°C or 26°C. Glu concentrations were 0.5 mM (yellow) or 1 mM (blue). Results are expressed as percentage inhibition of PR growth over the treatment period compared to controls without Glu i.e. [(PR growth in the absence of Glu − PR growth in the presence of Glu)/PR growth in the absence of Glu] × 100. Data from two experiments showing similar results have been combined (*n* = 11–12; ±SE). Dij-G, Dijon-G; L*er*, Landsberg *erecta*; Nd-0, Niederzenz. Different letters indicate statistically significant differences between groups based on one-way ANOVA, Tukey post-hoc, *P* = 0.05.

In preliminary experiments it was noted that the root response to Glu in Col-0 was surprisingly sensitive to relatively small differences in the growth temperature. To investigate this further and to determine its genotype dependence, the Glu sensitivities of five Arabidopsis accessions were compared at 20°C and 26°C (Fig. 4). At 20°C, PR growth in all five accessions was significantly inhibited by Glu, although to varying extents, consistent with previous evidence for the strong variation between accessions in their Glu sensitivity measured at 22°C (Walch-Liu *et al.*, 2006). However, in three of the five accessions the sensitivity to Glu declined markedly at 26°C. Col-0 was the most temperature-sensitive, almost completely losing its responsiveness to either 0.5 mM or 1 mM Glu at the higher temperature, while C24 was the least temperature-sensitive, its roots being strongly inhibited by Glu at both temperatures. Dijon-G and L*er* had an intermediate phenotype in which the Glu response at 26°C was partially reduced and Nd-0 showed the opposite effect, being slightly more responsive to Glu at the higher temperature. Thus, like the Glu sensitivity trait itself, the temperature sensitivity of the Glu response is highly dependent on genotype and therefore, as described below, is a potential target for QTL mapping the responsible loci.

### QTL mapping of Glu sensitivity loci under multiple environmental conditions

The exceptional environmental sensitivity of the Glu response is illustrated both by its temperature dependence (Fig. 4) and by the loss of Glu sensitivity that has previously been observed in the presence of excess nitrate (Walch-Liu and Forde, 2008). To investigate the genetic control of this environmental sensitivity, a series of experiments was performed in which 88 of the C24/Col-0 RILs were cultivated in growth cabinets under five different environmental conditions: minus nitrate at 20ºC, minus nitrate at 24ºC; minus nitrate at 26ºC; minus nitrate at 24°C with shading, and plus nitrate at 24ºC. The nitrate concentration used, 0.5 mM, was previously shown to strongly antagonise the root response to 50 µM Glu in C24 (Walch-Liu and Forde, 2007). An overlapping series of experiments was initiated at intervals over a period of 4 weeks and each environmental treatment was repeated once, resulting a total of 10 experiments. The frequency distribution plots (Supplementary Fig. S3), which are based on the combined data for all experiments, confirm the high degree of phenotypic variation for PR growth both PBT and PAT_Glu_ under all five conditions. For the most part the environmental conditions had only a minor effect on PR growth before the Glu treatment (PBT) in either the RIL population or the parental lines (Supplementary Fig. S3A). However, at 20°C the average PR length was markedly reduced compared to 24°C, and there was also a slight positive effect of the nitrate treatment. The data for PAT_Glu_ (Supplementary Fig. S3B) reveal a similar picture, except that the phenotypic variance in the RIL population was much greater than for PBT under all conditions. In addition, the positive effect of nitrate was much stronger on PAT_Glu_ than on PBT in both the C24 and RIL populations, reflecting the antagonistic effect of nitrate on Glu sensitivity in C24. Note that because Col-0 is not significantly inhibited by this low concentration of Glu, the antagonistic effect of nitrate on its Glu sensitivity was not detectable here. Transgressive segregation for the PAT_Glu_ trait was also particularly evident in the presence of nitrate, suggesting that alleles from both parents were contributing to nitrate antagonism of the Glu response.

**Fig. 5.**
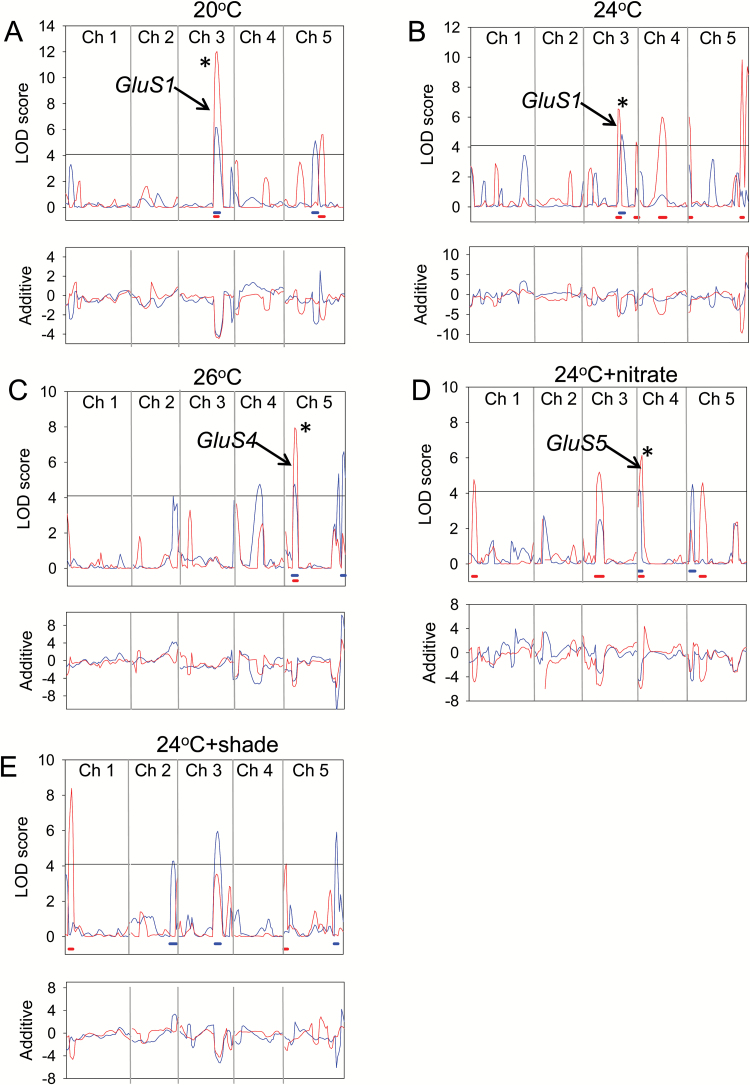
QTL analysis of Glu sensitivity (PAT_Glu_) in the Col-0/C24 RIL population grown under a range of environmental conditions. A set of 88 RILs was germinated and grown under a range of environmental conditions (A) 20°C (B) 24°C (C) 26°C (D) 24°C plus nitrate (E) 24°C with shade. In the absence of shading the light intensity was 330 µmol m^-2^ s^-1^ and under shade it was 130 µmol m^-2^ s^-1^. After 4 d, seedlings were transferred to fresh plates containing 50 µM K glutamate, with triplicate 12 × 12 cm plates with two RILs per plate and 2 seedlings of each RIL. Growth was continued for a further 5 d. Each experiment was performed in duplicate. PR growth after transfer to Glu (PAT_Glu_) was determined for each RIL, the data analysed using PLABQTL and the LOD scores plotted on the chromosome map. Red, first experiment; blue, second experiment. LOD significance thresholds (α = 0.05) were determined by 1000 permutations for each population and are shown as horizontal lines. The support intervals with a LOD fall-off of 1.0 for each significant QTL peak are shown as blue or red horizontal lines below the peaks. The lower graphs show plots of the additive effect of each region on the phenotype with respect to the C24 allele. Asterisks indicate where significant QTL peaks coincided in both experiments.

The LOD score plots obtained from composite interval mapping of the PBT and PAT_Glu_ traits in the multi-environment experiment are shown in Supplementary Fig. S4 and Fig. 5, respectively. Two complete datasets representing all five environmental conditions were analysed using the PLABQTL software, each comprising data from one of the two sets of replicates. The results indicate a strong genotype-by-environment interaction in the controls for the Glu response, with major differences in the loci controlling this trait under different environmental conditions. The major QTL on chromosome 3 that was identified at 21°C in the first experiment, *GluS1*, was confirmed in these experiments at both 20°C and 24°C. In each case it was the only QTL to be detected in both replicate experiments, contributing an average of 32% and 13.5% of the phenotypic variance for PAT_Glu_ at 20°C and 24°C, respectively. New QTLs were observed at 26°C, *GluS4* with both peak LOD scores at 14 cM on chromosome 5, and at 24°C plus nitrate, *GluS5* with peak LOD scores at 2 and 6 cM on chromosome 4. Again *GluS4* and *GluS5* were the only QTLs detected in both replicate experiments under these conditions, accounting for an average of 19% of the phenotypic variance in each case. No consistent QTL peaks for PAT_Glu_ were detected in the 24°C with shade treatment, but in one of the replicates there were peaks above the LOD threshold that colocalised with *GluS1* and *GluS3* on chromosomes 3 and 5, respectively (Fig. 3). As noted above for the QTLs detected in the first experiment, all of the newly detected QTL in this series of experiments were attributable to C24 alleles conferring enhanced sensitivity to Glu. Table 1 summarizes the QTLs whose LOD score exceeded the 5% threshold value in both replicates.

The environmental conditions also had strong effects on the QTLs identified for the PBT trait (Supplementary Fig. S4 and Table S3). The peak (*PBT1*) observed near the top of chromosome 1 in the initial experiment (Fig. 3) was only seen at 24°C plus nitrate in these experiments, with both peak LOD scores at 20 cM on chromosome 1. Three other QTLs for the PBT trait were observed in both replicate experiments: *PBT2* with peak LOD scores at 10 and 12 cM on chromosome 4 at 24°C only, *PBT3* with peak LOD scores at 24 and 26 cM on chromosome 5 at 24°C plus nitrate only and *PBT4* with peak LOD scores at 48 and 52 cM on chromosome 4 at 24°C with shade. Note that here we cannot distinguish between genotypic effects on germination time and effects on root elongation rate, since both will potentially affect the length of the PR at the time of transfer. Importantly, however, none of the QTLs identified for the PBT trait corresponded to those seen for PAT_Glu_.

### Confirmation and mapping of a major QTL for glutamate sensitivity using a set of Col-0/C24 ILs

**Fig. 6.**
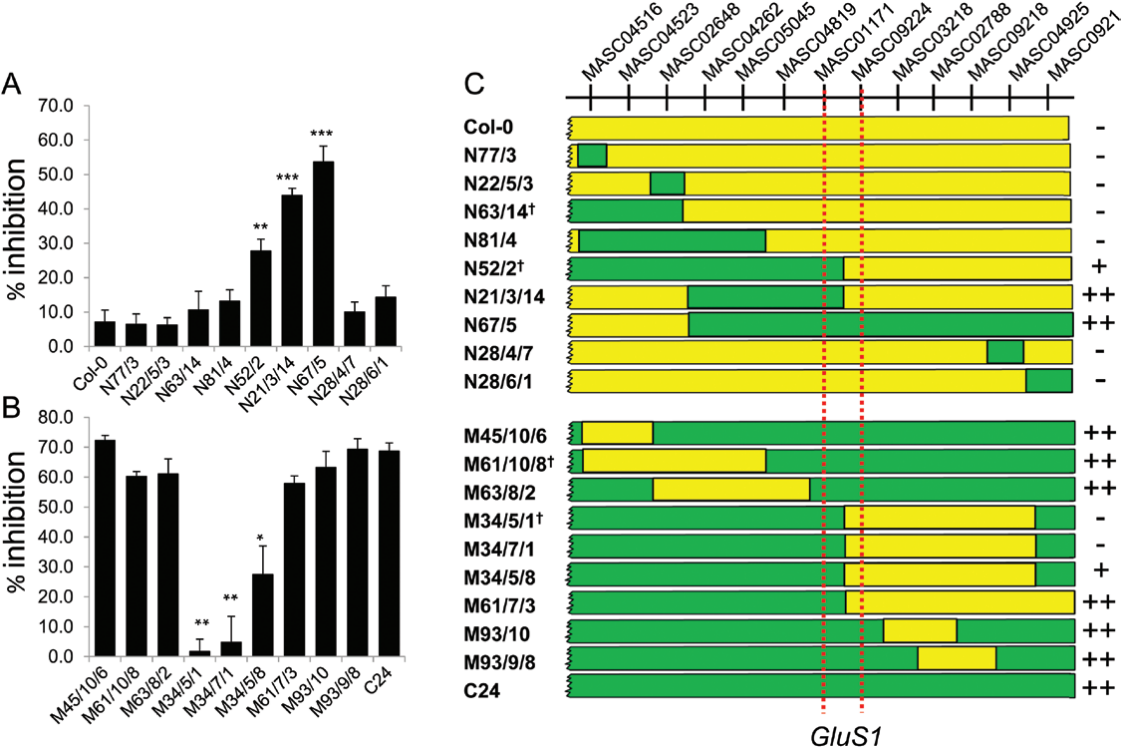
Use of introgression lines to identify the region of chromosome 3 carrying a major QTL for Glu sensitivity. Eighteen ILs, nine with a Col-0 background and nine with a C24 background, together with the Col-0 and C24 parental lines, were germinated and 4 d-old seedlings transferred to plates with and without 50 µM K glutamate. The increase in PR length over the following 5 d was measured and Glu sensitivity for each line expressed as percentage inhibition compared to controls. (A) Col-0 and nine Col-0 ILs with introgressions from C24 in chromosome 3 and (B) C24 and nine C24 ILs with introgressions from Col-0 in chromosome 3. Asterisks indicate those ILs where the Glu sensitivity differed significantly from the respective parental line based on a Student’s *t* test (** = *P* < 0.01; *** = *P* < 0.001; *n* = 9 ± SE); (C) Schematic showing the locations of introgressions in the eighteen ILs. Green, C24; yellow, Col-0. Note that only the bottom end of chromosome 3 is depicted. ILs indicated with a dagger have introgressions that are not fully represented in this schematic: in N63/14 the introgression from C24 extends to MASC03898 at the top of chromosome 3; in N52/2 the introgression from C24 extends upwards on chromosome 3 to MASC04279; M61/10/8 carries an additional introgression from Col-0 on chromosome 5 from MASC03559 to MASC01361 and M34/5/1 carries an additional introgression from Col-0 on chromosome 1 from MASC03658 to MASC02998 (Supplementary Table S4). Plus signs down the right hand side indicate ILs showing Glu hypersensitivity. The red dashed lines indicate the deduced extremities of the region carrying the *GluS1* locus whose C24 allele confers Glu hypersensitivity.

We employed a set of reciprocal Col-0/C24 ILs [(Törjek *et al.*, 2008) and Supplementary Table S4] to confirm and more accurately map *GluS1,* the major QTL for Glu sensitivity on chromosome 3. Glu sensitivity of PR growth was assayed at 20°C in seedlings of nine Col-0 ILs carrying introgressions from C24 in the relevant region of chromosome 3 and nine C24 ILs with Col-0 introgressions in the same region. The map positions of the introgressions and the results of the Glu sensitivity assay are shown in Fig. 6. Six of the ILs in the Col-0 background resembled Col-0 in being almost insensitive to 50 µM Glu. However the other three, N52/2, N21/3/14 and N67/5, showed greatly increased sensitivity. Amongst the C24 ILs, seven lines were similar to the C24 parent, but two lines, M34/5/1 and M34/7/1, had almost completely lost Glu sensitivity and one line, M34/5/8, had intermediate sensitivity. The map locations of the introgressions in these 18 ILs define the region of chromosome 3 in C24 responsible for conferring Glu sensitivity as located between framework markers MASC01171 and MASC09224 (Fig. 6C). These markers are located at 57.1 and 67.7 cM on chromosome 3, respectively, which corresponds well with the location of *GluS1* close to MASC01171 as determined by several QTL mapping experiments (Figs 3 and 5, summarized in Table 1).

Six of the ILs, N52/2, N21/3/14, M61/7/3, M34/5/1, M34/7/1 and M34/5/8, were of particular interest because their introgressions begin or end within the region of interest. The images in Supplementary Fig. S5 show the extent to which these introgressions affected how the roots responded to Glu. Supplementary Fig. S6 shows how more detailed mapping of these lines allowed the *GluS1* locus to be located to the interval between the At3g44830 and At3g46880 genes.

### Using ILs to locate loci conferring temperature sensitivity on the root response to Glu

To investigate the genetic control of temperature sensitivity of the Glu response in more detail, a set of 32 ILs in the C24 background were assayed for their sensitivity to 50 µM Glu at three temperatures: 20°C, 24°C and 26°C. These 32 ILs carry introgressions from Col-0 that collectively cover around 80–90% of the genome (Törjek *et al.*, 2006). The data are plotted in Supplementary Fig. S7 in the form of reaction norms. The ILs have been divided into four groups according to their Glu sensitivity at 20°C and the responsiveness of this trait to temperature. Group 1 (Supplementary Fig. S7A) is the largest group and contains 13 ILs that resembled the C24 parent in being both hypersensitive to Glu and having low sensitivity to the increase in temperature, with 64–85% inhibition by Glu at 26°C. The nine ILs in Group 2 (Supplementary Fig. S7B) are similarly hypersensitive to Glu but show a moderate degree of temperature sensitivity, with 43–61% inhibition by Glu at 26°C. The six ILs in Group 3 (Supplementary Fig. S7C) are characterized by an already diminished sensitivity to Glu at 20°C combined with only minimal responsiveness to temperature. The four ILs in Group 4, M100/2/9/5, M31/8, M48/5/1 and M97/1/6, stand out as having acquired strong temperature sensitivity while maintaining the Glu hypersensitivity of the C24 background, showing >80% inhibition at 20°C and just 4–37% inhibition at 26°C (Supplementary Fig. S7D). Only two chromosomal regions are represented in these four ILs, one at the top of chromosome 1 and the other on the long arm of chromosome 5. M100/2/9/5 carries an introgression on chromosome 1 that includes framework markers MASC03771–MASC09203. However the lack of clear temperature sensitivity in two other ILs, M82/1/2 and M65/6/6, with introgressions at framework markers MASC03771 and MASC03758–MASC05303, respectively, indicates that the relevant locus that we designate *TS1* lies between markers MASC03771 and MASC03758 (genes At1g01471-At1g07520). The second temperature sensitivity locus (*TS2*), is defined by the introgressions in M31/8 (MASC04983–MASC04350), M48/5/1 (MASC04591– MASC04576) and M97/1/6 (MASC03559 plus MASC01545–MASC04350), as being between markers MASC02675 and MASC09211 (genes At5g49680–At5g63920). This locus overlaps with the Glu sensitivity QTL *GluS3*.

**Fig. 7.**
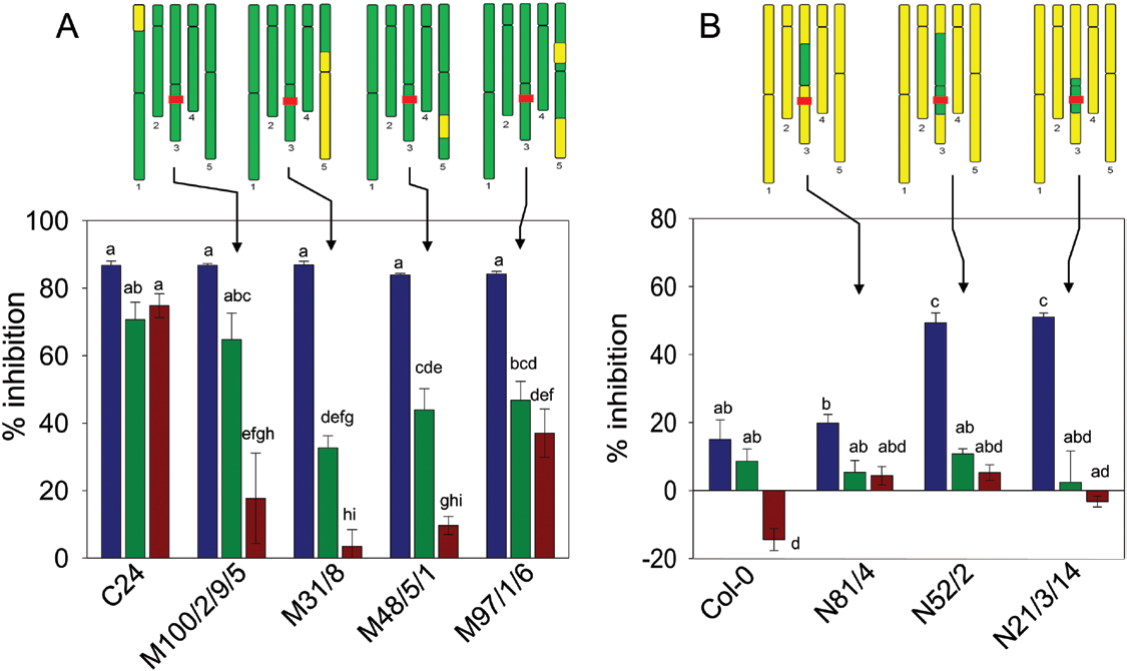
Use of introgression lines to map QTLs controlling the temperature sensitivity of the Glu effect. For each IL, 4 d-old seedlings were transferred to agar plates with and without 50 µM Glu. Seedling growth before and after transfer was at 20°C (blue), 24°C (green) or 26°C (red). The increase in PR length over the following 5 d was measured and Glu sensitivity for each line is expressed as percentage inhibition compared with controls. (A) C24 and the four C24 ILs with C24-like sensitivity to Glu at 20°C and markedly reduced Glu sensitivity at 26°C. (B) Col-0 and three Col-0 ILs with introgressions in chromosome 3. Different letters indicate statistically significant differences between groups based on one-way ANOVA, Tukey post-hoc, *P* = 0.05, *n* = 6 ± SE. The schematics above each graph indicate the approximate locations of the introgressions on the five chromosomes. Green, C24; yellow, Col-0. The horizontal red line indicates the approximate location of the *GluS1* locus.

It is striking that all five C24 ILs in which the region of chromosome 3 containing the *GluS1*^*C24*^ allele has been replaced by the equivalent region from Col-0 (M34/7/1, M28/11/2, M34/5/8, M34/5/1, M28/11/1) fell into Group 3 i.e. they showed the same low degree of temperature sensitivity as C24 itself (Supplementary Fig. S7C). This indicates that the *GluS1*^*Col-0*^ allele is not intrinsically temperature sensitive and that epistatic interactions with other loci in the Col-0 genome are probably responsible for its temperature-sensitive phenotype. This was confirmed by the reciprocal introgressions, where the region containing the *GluS1*^*Col-0*^ allele was replaced by the *GluS1*^*C24*^ allele in two Col-0 ILs, N52/2 and N21/3/14. These lines were, as expected, much more sensitive to Glu than Col-0 at 20°C but this sensitivity was lost at 24°C and 26°C (Fig. 7B), indicating that temperature sensitivity had been conferred on the *GluS1*^*C24*^ allele by its presence in the Col-0 background. It seems likely that the Col-0 alleles at the *TS1* and *TS2* loci, on chromosomes 1 and 5, respectively, are at least partly responsible for these epistatic effects.

### Using ILs to locate loci conferring nitrate sensitivity on the root response to glutamate

**Fig. 8.**
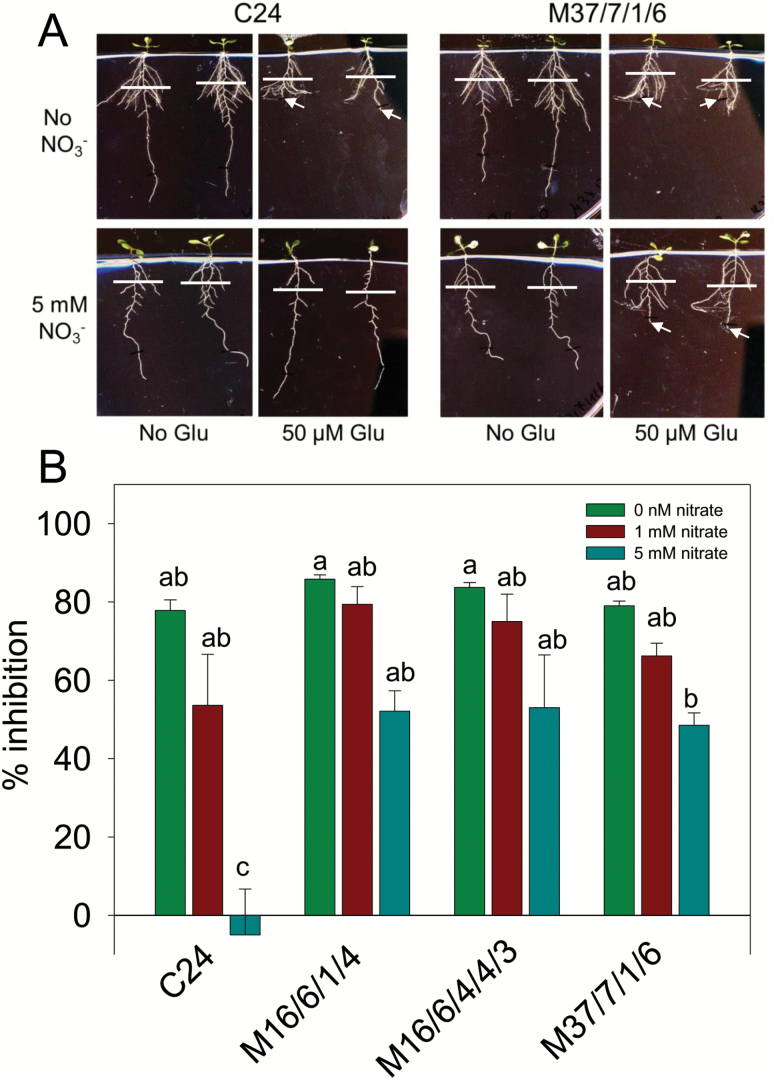
Identification of a QTL on chromosome 1 controlling nitrate sensitivity of the Glu effect. (A) Four d-old seedlings of introgression line M37/7/1/6 and its C24 parental line were transferred to agar plates containing medium with or without 5 mM KNO_3_ in the presence or absence of 50 µM Glu. Seedlings were imaged after a further 5 d. White lines mark the positions of the PR tips at the time of transfer. Note that where PRs were strongly inhibited by Glu it was sometimes necessary to move adjacent lateral roots aside to reveal the PR tip (arrowed) before imaging. (B) Seedlings of C24 and three ILs carrying overlapping introgressions on the long arm of chromosome 1 were transferred to agar plates containing 0 mM, 1 mM or 5 mM KNO_3_ in combination with either no Glu or 50 µM Glu. For each nitrate concentration, percentage inhibition of PR growth in the 5 d after transfer to Glu was calculated relative to roots treated with the same nitrate concentration without Glu (± SE; *n* = 6). Different letters indicate statistically significant differences between groups based on one-way ANOVA, Tukey post-hoc, *P* = 0.05.

The C24/Col-0 ILs were also used to identify loci responsible for controlling nitrate antagonism of the Glu response. When 32 C24 ILs were grown on medium with and without Glu in the presence and absence of nitrate there was a wide and continuous variation in the degree to which nitrate was able to overcome the Glu effect. This is illustrated by percentage inhibition by Glu in the absence of nitrate plotted against percentage inhibition in the presence of nitrate (Supplementary Fig. S8). This indicates that there are probably multiple genes of small effect contributing to this response to nitrate. However, there were several ILs that were notable for being as sensitive as C24 to Glu in the absence of nitrate but, unlike C24, lost little of this sensitivity in the presence of nitrate (Supplementary Fig. S8). The line that showed the least sensitivity to nitrate was M37/7/1/6 and the images in Fig. 8A illustrate the striking difference in root system architecture between it and C24 when seedlings of each genotype were grown on Glu in the presence of nitrate.

Another of the lines showing greatly diminished sensitivity to nitrate was M16/6/1/4, which carries an introgression on chromosome 1 that overlaps with the one in M37/7/1/6 (Supplementary Table S4). Both these lines were therefore analyzed in more detail, alongside an additional IL with an introgression in the same region, M16/6/4/4/3, by growing them on medium with and without Glu and with nitrate concentrations of 0 mM, 1 mM or 5 mM. The data in Fig. 8B confirm the low sensitivity of all three lines to 5 mM nitrate compared to C24. Only the chromosomal segment carrying markers MASC03631–MASC03684 is common to all three ILs, indicating that the introgressed locus responsible for the reduction in nitrate sensitivity, designated *NS1*, is located between markers MASC03447 and MASC03930 (genes At1g64680-At1g74045). However, another C24 IL with an introgression spanning markers MASC03631 and MASC03684, M37/7/8/6, showed normal sensitivity to nitrate (Supplementary Fig. S8), indicating that within this region of interest we are able to exclude the genes located in the interval between genes At1g67350 and At1g70220. M63/9/3, which carries an introgression at the top of chromosome 4 spanning markers MASC04123–MASC04685, showed a loss of nitrate sensitivity similar to M16/6/1/4 and M37/7/1/6 (Supplementary Fig. S8). However this locus was not investigated in more detail.

## Discussion

### Identification of a major QTL for Glu sensitivity on chromosome 3

It was previously established that there are marked differences between Arabidopsis accessions in the Glu sensitivity of their PR growth, the most sensitive of those tested being C24 and one of the least sensitive being Col-0 (Walch-Liu *et al.*, 2006). Using a combination of RILs and ILs from a Col-0/C24 population it has now been possible to identify several QTLs that significantly contribute to the Glu hypersensitivity of C24. The RIL mapping experiments (Figs 3 and 5) identified one large-effect QTL, *GluS1*, and two less prominent QTLs, *GluS2* and *GluS3*, which together accounted for up to 42% of the phenotypic variance depending on the experimental conditions. In all three QTLs, Glu sensitivity was conferred by the C24 genome, suggesting a history of strong selection for these alleles in this accession.

The contribution of *GluS1* to Glu sensitivity was dependent on environmental conditions (Fig. 5), but in most experiments it was either the sole or the major QTL detected, contributing up to 32% of the variation in Glu sensitivity in the RIL population (Table 1). ILs in the C24 background carrying Col-0 introgressions in the region of MASC01171 and reciprocal Col-0 ILs carrying C24 introgressions in the same region, were used to demonstrate that this C24 locus, *GluS1*^C24^, was not only required for the Glu hypersensitivity phenotype in C24 but was also sufficient to confer Glu hypersensitivity when introgressed into Col-0 (Fig. 6).

Based on the six RIL mapping experiments in which the QTL was detected (summarised in Table 1), the mean location of the *GluS1* peak on chromosome 3 was at 57.3 cM, placing it very close to MASC01171 located at 57.1 cM. This was confirmed from analysis of the Glu sensitivities of a series of available ILs with introgressions in this region (Fig. 6 and Supplementary Fig. S6), which showed that the *GluS1* locus is located much closer to MASC01171 than to MASC09224 and, more specifically, that it lies between genes At3g44830 and A3g46880. Of the 200 or so genes in this interval, one noteworthy candidate is At3g45640, which encodes MAP kinase 3 (MPK3). MPK3 is known to be involved in defence signaling (Pitzschke *et al.*, 2009). However a recent report indicated that it is a component of a MAP kinase signaling cascade downstream of MAP kinase kinase 9 (MKK9), which is involved in regulating the physiological response to low phosphate (Lei *et al.*, 2014). Mutants at the *MPK3* locus had shorter PRs and higher lateral root densities than Col-0 under both low and high phosphate conditions, indicating effects on root growth and development that are independent of phosphate status (Lei *et al.*, 2014). A previous study revealed an important positive role for the MEKK1 MAP kinase kinase kinase in controlling the root’s sensitivity to Glu (Forde *et al.*, 2013). Despite earlier evidence for a role of MEKK1 in a signaling cascade leading to activation of MPK3 (Asai *et al.*, 2002), it is no longer clear whether MEKK1 is required for activation of this pathway, at least in the case of the flg22-elicited defence response (Suarez-Rodriguez *et al.*, 2007). Nevertheless it remains possible that MEKK1 and MPK3 are part of the same signaling cascade by which Glu inhibits root growth. Note that MEKK1 (At4g08500) maps to ~10 cM on chromosome 4 (Supplementary Table S5) and therefore does not co-locate with any QTLs identified in this study.

Potential candidates for a role in Glu-elicited effects on root growth include the family of 20 glutamate receptor-like (*GLR*) genes that encode amino acid activated Ca^2+^ channels (Dietrich *et al.*, 2010). A defect in a rice *GLR* gene led to loss of meristematic activity in the root tip, suggesting a role in maintaining the integrity of the root apical meristem (Li *et al.*, 2006). Of the QTLs identified in this study, only one maps close to any members of the Arabidopsis *GLR* (*AtGLR*) family. The marker most closely linked to *GluS4*, which was only detected at 26°C, is MASC05127. It is located within At5g13260, so *GluS4* overlaps with the nearby *AtGLR2.5* (At5g11210) and *AtGLR2.6* (At5g11180) genes. Like all other *GLR* genes, both *AtGLR2.5* and *AtGLR2.6* are expressed in roots (Chiu *et al.*, 2002; Roy *et al.*, 2008), but their function is unknown.

### *QTLs for primary root growth in the absence of glutamate were distinct from the* GluS *loci*

Four QTLs for PR growth in the absence of Glu i.e. in the period before Glu treatment started, were identified (*PBT1-4*; Supplementary Table S3). Like the *GluS* QTLs, all the *PBT* QTLs were sensitive to the environment, none being found consistently across all conditions tested. Importantly, however, none of the *PBT* loci co-locate with any of the *GluS* loci, showing that the QTLs responsible for differences in PR growth in the absence of Glu are distinct from those responsible for variations in growth rate in the presence of Glu.

*PBT1* is in the same region of chromosome 1 as *LPR1*, a QTL for the PR growth response to low phosphate that was originally mapped in a Bay-0 × Shahdara population (Reymond *et al.*, 2006). The *PBT1* peak was located between 16 and 22 cM (Supplementary Table S3), while *LPR1* was mapped at ~18 cM on chromosome 1 and has been identified as At1g23010, which encodes a multicopper oxidase (Svistoonoff *et al.*, 2007). The low phosphate concentration of 22 µM in our growth medium would be consistent with detection of this QTL. In addition, *PBT4*, mapped at ~50 cM on chromosome 4, which was detected only in plants grown in low light conditions at 24°C, maps in the same region of chromosome 4 as *LPR3* at 50.8 cM. *LPR3* is a QTL for root growth in the same population whose effect was independent of the phosphate supply (Reymond *et al.*, 2006). *LPR3* also co-locates with *PRL3* at 52.1 cM, a QTL for PR growth mapped in the same set of Bay-0 × Shahdara RILs (Loudet *et al.*, 2005).

### Temperature sensitivity of the Glu effect is conferred epistatically by at least two separate loci

The environmental sensitivity of the Glu effect was previously demonstrated by the ability of excess nitrate to suppress it (Walch-Liu and Forde, 2008). This paper supports these findings through the identification of Arabidopsis accessions where Glu sensitivity was strongly influenced by the prevailing temperature, even over a relatively narrow temperature range of 20°C to 26°C (Fig. 4). Why there should be such a strong interaction between temperature and Glu sensitivity is unknown, but it may be significant that C24, the accession that was least sensitive to temperature, is also the one that originates from the most southerly latitude of 40.2°N in Portugal, whereas Col-0, L*er*-0, Dijon-G and Nd-0 are all from Central Europe with latitudes in the range of 47–52°N (Schmid *et al.*, 2006). A study of the temperature sensitivity of petiole hyponasty found that C24 and other accessions from nearer the equator were less responsive to high temperatures than accessions from more northern latitudes, such as L*er* and Col-0 (van Zanten *et al.*, 2009), although the temperatures involved were much higher and exposure much briefer than here i.e. 7 h at 38°C.

Further evidence of the environmental sensitivity of the Glu effect can be seen in how the environmental conditions of temperature, light and nitrate, affected which *GluS* QTLs were detected, with none being common to all conditions (Fig. 5 and Table 1). Similar interactions between the environment and QTL effects have previously been reported for flowering time variation in Arabidopsis (Stratton, 1998; Weinig *et al.*, 2002).

The contrasting temperature sensitivities of C24 and Col-0 prompted us to use the Col-0/C24 ILs to investigate the genetic control of this phenomenon. Five C24 ILs carried introgressions that replaced the region of chromosome 3 containing the major Glu sensitivity locus, *GluS1*, with the corresponding region from Col-0. In each case their temperature sensitivity was found to resemble that of C24 rather than Col-0 (Supplementary Fig. S7C). Furthermore, the reciprocal lines where the *GluS1*^*C24*^ allele was introduced into the Col-0 background showed Col-0-like temperature-sensitivity (Fig. 7B). Thus it appears that temperature sensitivity is conferred on the *GluS1* locus by genes elsewhere in the Col-0 background. Two candidate epistatic loci, *TS1* and *TS2*, were identified on the basis that Col-0 introgressions from these regions in the C24 background conferred strong temperature sensitivity (Supplementary Fig. S7D). *TS1* was provisionally mapped to the region between genes At1g01471 and At1g10560, and *TS2* to the region between At5g49680 and At5g63920 overlapping with *GluS3*. Ambient temperature signaling has received less attention than signaling related to extremes of heat and cold. Nevertheless, a number of mechanisms for sensing smaller shifts in temperature have been proposed, including RNA folding, protein-protein interactions, epigenetic effects and changes in membrane fluidity (Lee *et al.*, 2008). One candidate gene located within the *TS1* interval is *LONG HYPOCOTYL IN FAR-RED LIGHT1* (*HFR1*; At1g02340), which encodes a bHLH protein that has been identified as a component of a regulatory circuit, along with PHYTOCHROME INTERACTING FACTOR4 (PIF4), which is responsible for maintaining plant growth at high ambient temperatures of 25°C (Foreman *et al.*, 2011).

### Identification of a QTL controlling nitrate sensitivity of the Glu effect

It has been found that there are highly specific interactions between nitrate and Glu signalling at the Arabidopsis root tip. If the PR tip is simultaneously exposed to excess nitrate, but not other forms of N, then the inhibitory effect of Glu on root growth can be completely suppressed (Walch-Liu and Forde, 2008). The antagonistic effect of the NO_3_^-^ ion was shown to be dependent on the functionality of the *NPF6.3* (*NRT1.1/CHL1*) gene, probably in its role as a nitrate sensor (Walch-Liu and Forde, 2008). It has been suggested that the antagonistic interactions between nitrate and Glu signalling at the root tip could enable plants to modify their root architecture in response to changes in the relative abundance of the two forms of N, acting as cues for the inorganic and organic N pools in the soil (Walch-Liu and Forde, 2008).

Using the C24/Col-0 ILs to further investigate the genetic control of nitrate’s effect on Glu signaling, we found evidence for multiple loci affecting nitrate sensitivity. One locus found on the long arm of chromosome 1 had a particularly strong effect (Fig. 8). This region includes a cluster of four genes belonging to the same family as the NRT1 nitrate transceptor: At1g72120 (*NRT1.15/AtNPF5.14*), At1g72125 (*NRT1.16/AtNPF5.*13), At1g72130 (*AtNPF5.11*), At1g72140 (*AtNPF5.12*). The NPF family in Arabidopsis has 53 members belonging to 8 subfamilies and between them they transport a diverse range of substrates, including peptides and hormones, in addition to nitrate (Léran *et al.*, 2014). AtNPF5.13 and AtNPF5.14 are both reported to act as nitrate transporters when expressed in Xenopus oocytes (Tsay *et al.*, 2007). However, none of the four closely related genes in this cluster belongs to the same NPF subfamily as *NRT1.1* and none has previously been implicated in nitrate sensing.

## Conclusions

**Fig. 9.**
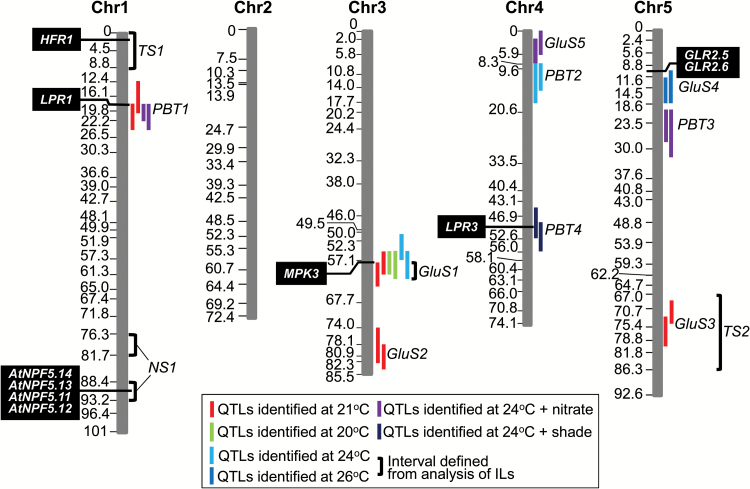
Diagram showing the map locations of the QTL and relevant candidate genes identified in the present study. For each of the nine QTL, *GluS1* to *GluS5* and *PBT1* to *PBT4*, the relevant LOD support intervals have been plotted alongside the chromosomal maps and colour-coded according to which of the six experiments they were detected in (see key, Table 1 and Supplementary Table S5). Also shown are the locations of the chromosomal intervals (*GluS1*, *TS1*, *TS2* and *NS1*) detected from analysis of the collection of C24/Col-0 ILs and referred to in the text. Candidate genes that co-locate with the QTLs and that are discussed in the text are shown as white text on a black background. The genetic map, in cM, is as previously generated from the reciprocal Col-0 × C24/C24 × Col-0 RIL population (Törjek *et al.*, 2006).

The high degree of natural variation in Glu sensitivity in Arabidopsis roots, together with the extent to which it is affected by the environmental factors of temperature, light and nitrate availability, has made it possible to use QTL analysis to gain new insights into the genetic control of these traits. Fig. 9 summarises the results of our mapping experiments in which RILs and ILs generated from Col-0 × C24 crosses were cultivated under a range of environmental conditions. These studies have led to the identification of three QTL, *GluS1*, *GluS2* and *GluS3*, which in combination can account for up to 42% of the phenotypic variance for Glu sensitivity in the RIL population, but whose contribution is strongly dependent on the environment. One QTL, *GluS1*, is particularly notable for its ability to confer Glu hypersensitivity when introgressed into a background with low Glu sensitivity. Other QTL were identified that are involved in conferring either temperature sensitivity (*TS1* and *TS2*) or nitrate sensitivity (*NS1*) on the Glu response. Thus we have obtained a first picture of the multiplicity of loci responsible for modulating the Glu sensitivity trait and their epistatic interactions. Further research will be needed to establish the identity of the genes that underlie these QTLs, beginning with the candidate genes identified above, and to understand the physiological relevance of the remarkable environmental sensitivity of the root’s response to Glu.

## Supplementary data

Supplementary data are available at *JXB* online.

Table S1. Composition of basal medium.

Table S2. CAPS and dCAPS primers used for fine mapping the *GluS1* locus.

Table S3. Summary of the QTL controlling the PBT trait.

Table S4. Map locations of introgressions in the ILs used in the present study.

Table S5. Physical and genetic map positions of framework SNP markers.

Fig. S1. Plots of ranked means and standard errors for the PBT data from the Col-0xC24 and C24xCol-0 RIL populations.

Fig. S2. Plots of ranked means and standard errors for the PAT_Glu_ data from the Col-0xC24 and C24xCol-0 RIL populations.

Fig. S3. Frequency distribution plots for PR growth of a RIL population in a multi-environment experiment ± Glu.

Fig. S4. QTL analysis of the PBT trait from a RIL population grown under a range of environmental conditions.

Fig. S5. Effect of a series of introgressions in the vicinity of the *GluS1* locus on Glu sensitivity.

Fig. S6. Fine mapping of the introgressions in six ILs used to define the position of the *GluS1* locus.

Fig. S7. Reaction norm plots for the effect of temperature on the Glu sensitivity of a set of C24 ILs with introgressions from Col-0.

Fig. S8. Scatter plot showing the effect of nitrate on the Glu sensitivity of a set of ILs in the C24 background.

## Supplementary Material

supplementary_figures_S1_S8

supplementary_tables_S1_S5

## Abbreviations:

CAPS: cleaved amplified polymorphism
Glu: L-glutamate
GluS1: Glu Sensitivity 1
IL: introgression line
LOD score: log of the odds score
PAT: primary root length after transfer
PBT: primary root length before transfer
PR: primary root
QTL: quantitative trait locus
RIL: recombinant inbred line
SNP: single nucleotide polymorphism.
