# Supplementary material for: QTL analysis of the developmental response to L-glutamate in Arabidopsis roots and its genotype-by-environment interactions
Source: J Exp Bot. 2017 Apr 26;68(11):2919–31. doi: 10.1093/jxb/erx132 (PMC5853333; doi:10.1093/jxb/erx132)

Fig. S1. Plots of ranked means and standard errors for the PBT data from the Col-0xC24 (A) and C24xCol-0 (B) RIL populations that were used for the QTL analysis shown in Fig. 3.

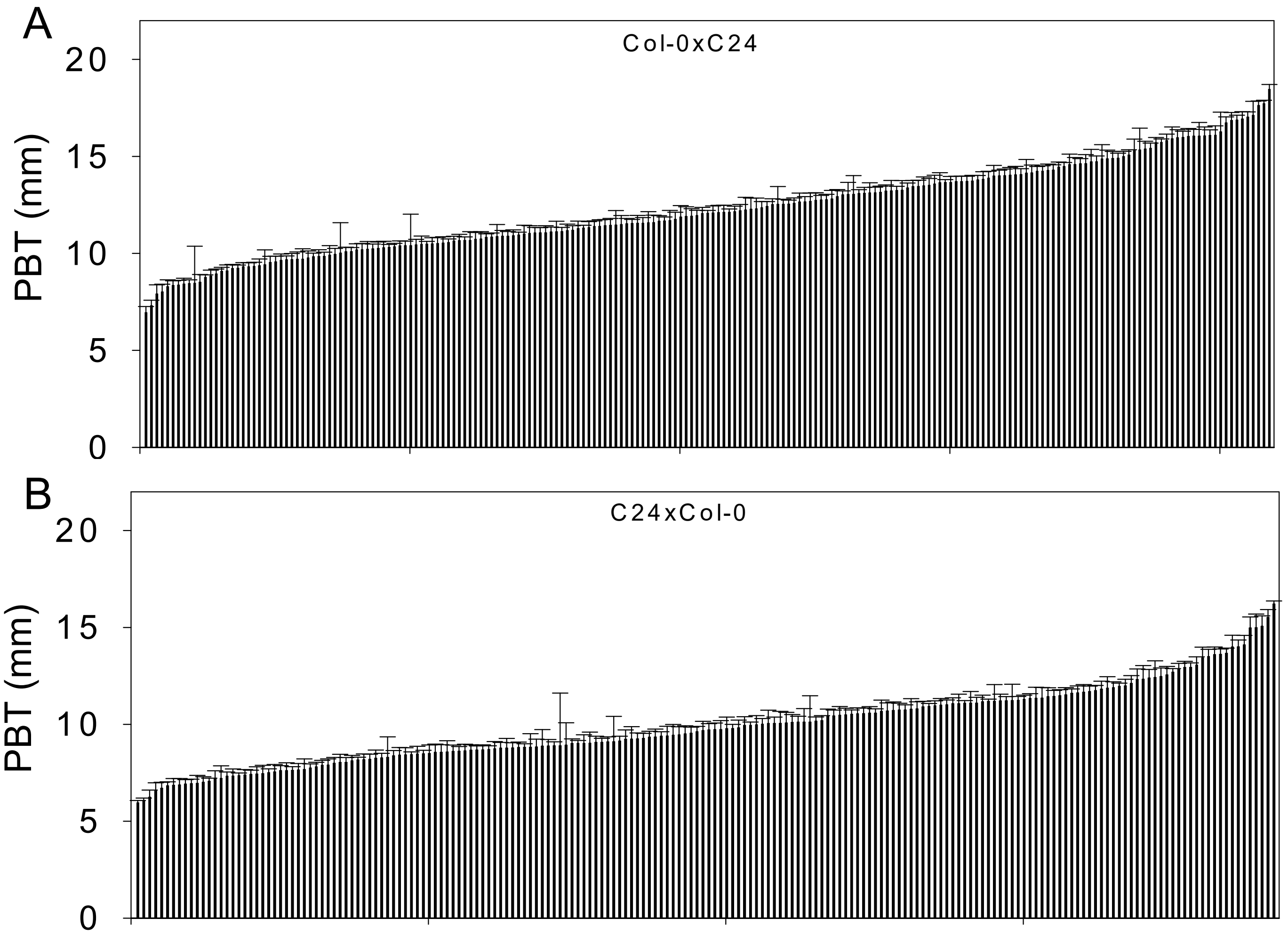

Fig. S2. Plots of ranked means and standard errors for the PAT_{Glu} data from the Col-0xC24 (A) and C24xCol-0 (B) RIL populations that were used for the QTL analysis shown in Fig. 3.

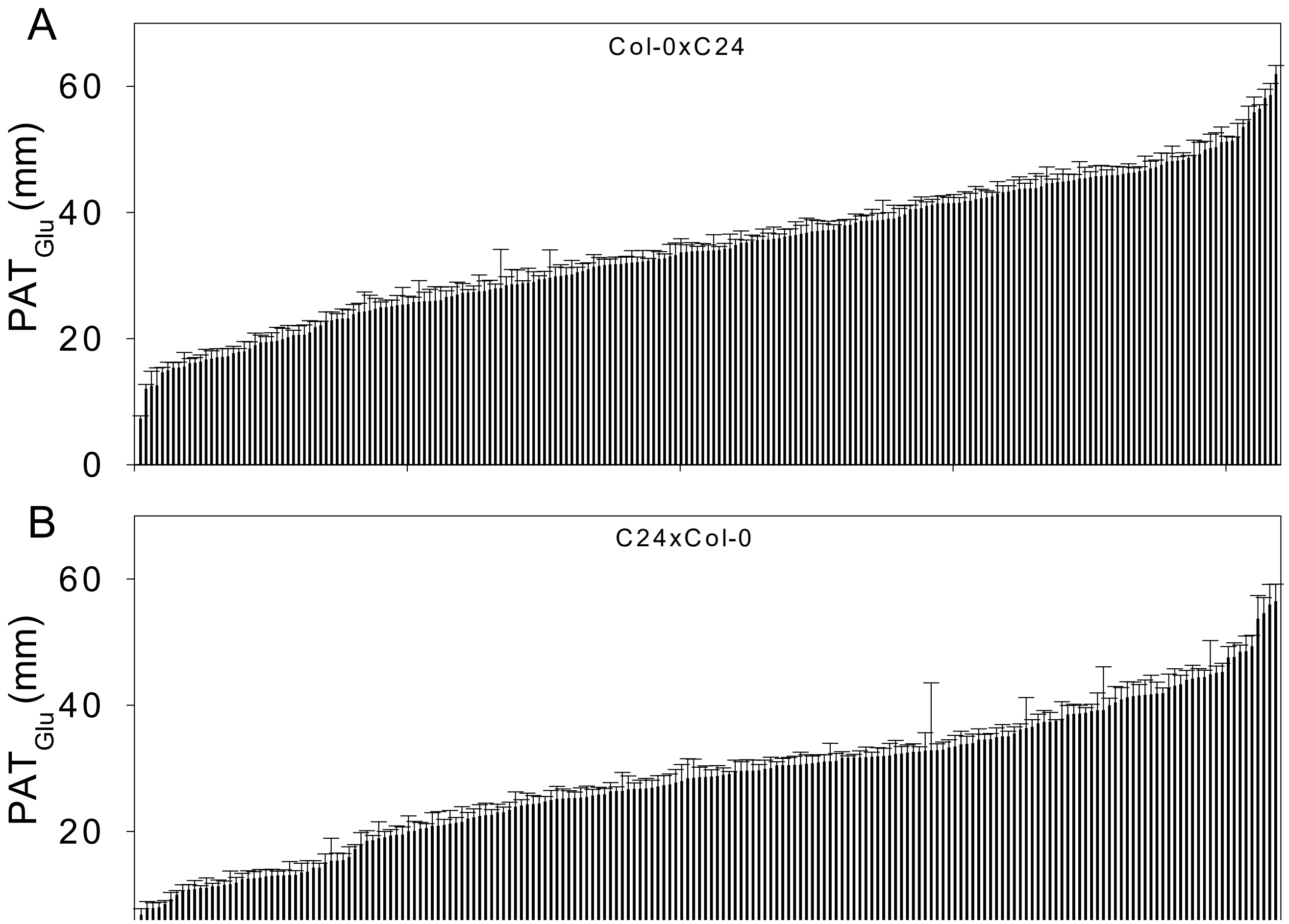

Fig. S3. Frequency distribution plots for primary root growth of 88 C24/Col-0 RILs in a multi-environment experiment before and after transfer to Glu. A set of 88 RILs (6 seedlings per line) were germinated and grown under a range of environmental conditions (20°C, 24°C, 26°C, 24°C+nitrate and 24°C+shade; see legend to Fig. 5 for further details). Each of the five sets of environmental conditions was applied from the time of germination and maintained during the period of treatment with 50 µM Glu. The frequency distribution plots are based on the combined data from duplicate experiments. (A) Length of the primary root before transfer (PBT). (B) Growth of the primary root after transfer to Glu (PAT^{Glu}). The overall means for the RIL population (± SD) are indicated in each case.

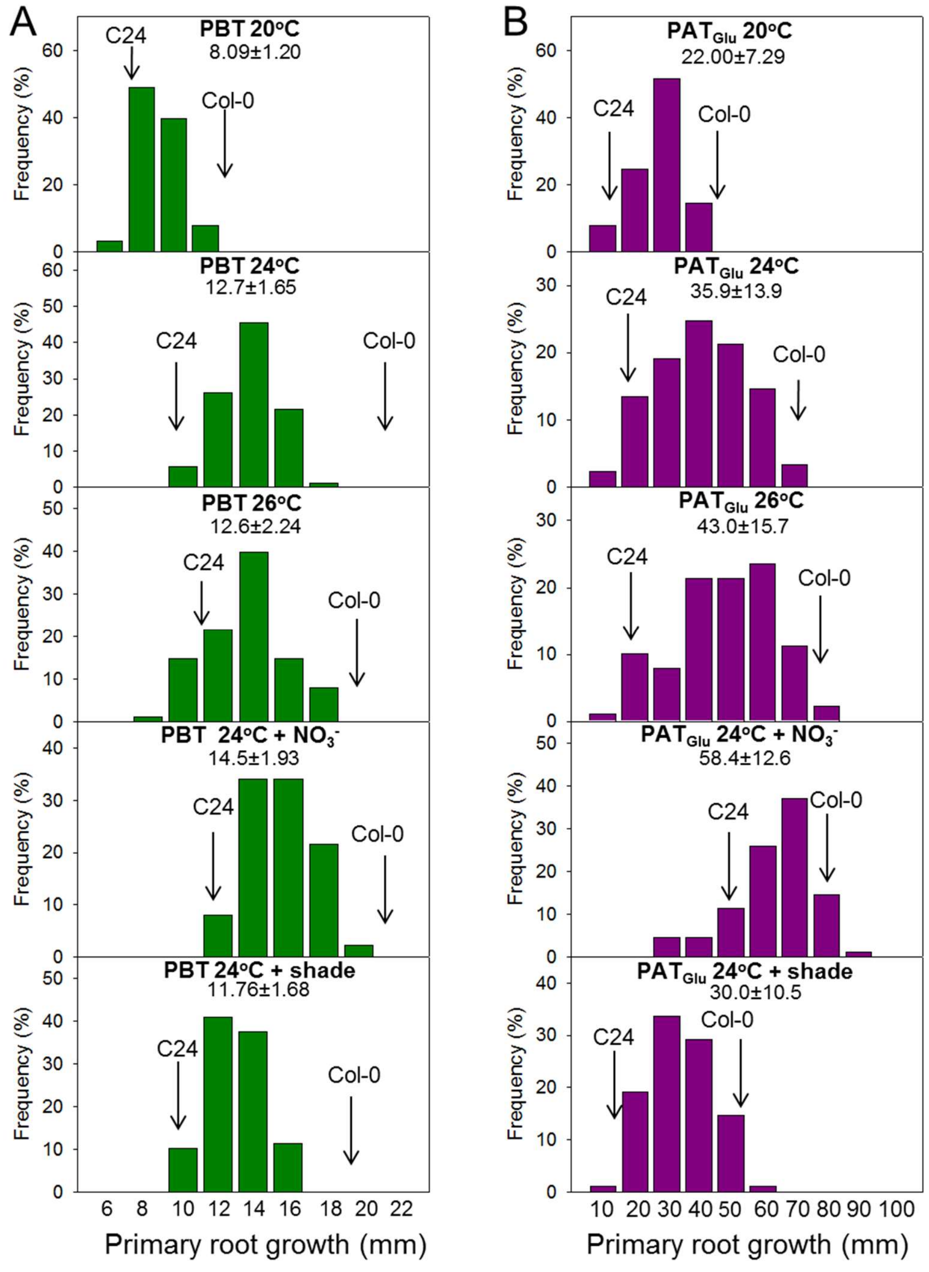

Fig. S4. QTL analysis of primary root growth before transfer (PBT) in the Col-0/C24 RIL population grown under a range of environmental conditions. A set of 88 RILs were germinated and grown under a range of environmental conditions (A) 20°C (B) 24°C (C) 26°C (D) 24°C+nitrate (E) 24°C+shade. Each experiment was performed in duplicate and other experimental details are set out in the legend to Fig. 5. Primary root growth before transfer (PBT) was determined for each RIL and the data were analysed using PLABQTL and the LOD scores plotted on the chromosome map (red = first experiment; blue = second experiment). LOD significance thresholds (α = 0.05) were determined by 1000 permutations for each population and are shown as horizontal lines. The support intervals (with a LOD fall-off of 1.0) for each significant QTL peak are shown as blue or red horizontal lines below the peaks. The lower graphs show plots of the additive effect of each region on the phenotype (with respect to the C24 allele). Asterisks indicate where significant QTL peaks coincided in both experiments.

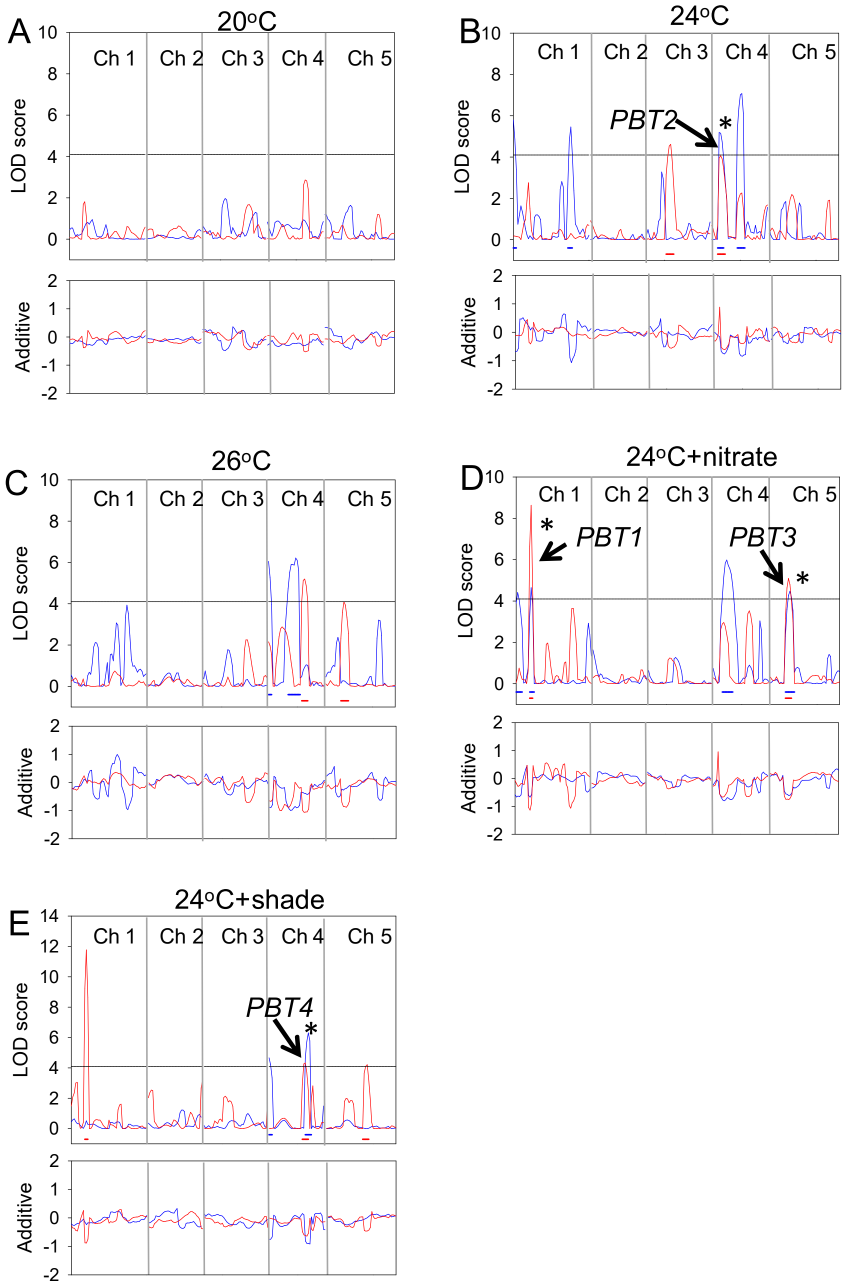

Fig. S5. Effect of a series of introgressions in the vicinity of the *GluS1* locus on glutamate sensitivity*.* Four d-old seedlings of each line were transferred to agar plates containing medium with no glutamate (-) or with 50 µM glutamate (Glu) and imaged after a further 5 d. White lines mark the positions of the primary root tips at the time of transfer. Note that where primary roots were strongly inhibited by glutamate it was sometimes necessary to move adjacent lateral roots aside to reveal the primary root tip (arrowed). before imaging (A) C24 and four ILs in the C24 background. (B) Col-0 and two ILs in the Col-0 background. Numbers in brackets indicate the framework markers mapping the introgressions.

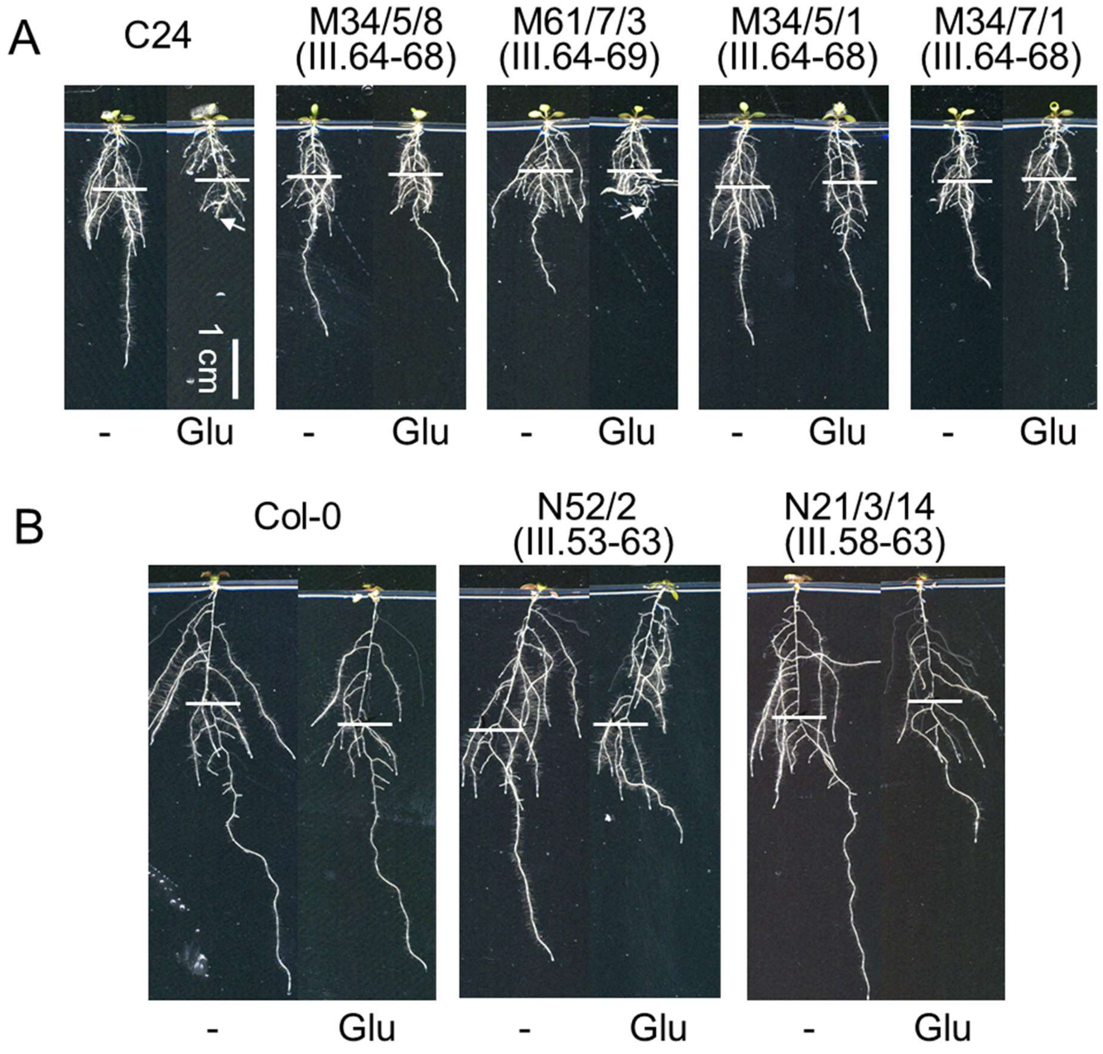

Fig. S6. Fine mapping of the introgressions in six ILs used to define the position of the *GluS1* locus. The genotypes of each IL at seven loci between MASC01171 and MASC09224 were determined using dCAPs or CAPs markers (see Table S1). Green = C24; yellow = Col-0. Lines with enhanced glutamate sensitivity (compared to Col-0) are indicated by + signs. The region deduced to contain the *GluS1* locus is bracketed.

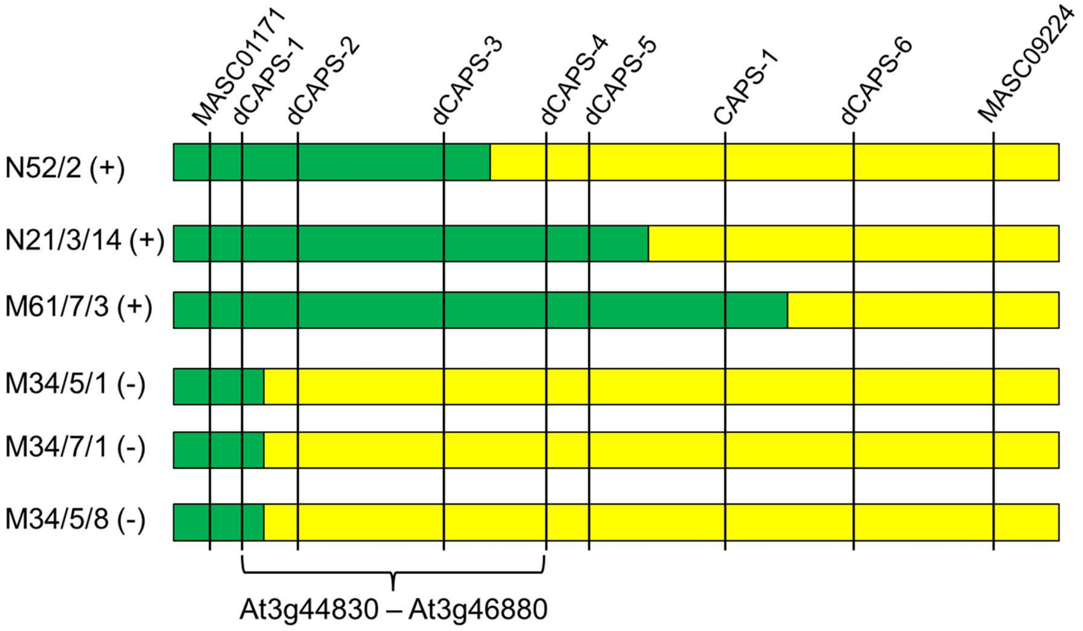

Fig. S7. Reaction norm plots for the effect of temperature on the glutamate sensitivity of a set of C24 ILs with introgressions from Col-0. Seedlings of each line were germinated and after 4 d transferred to fresh plates with and without 50 µM Glu at 20°C, 24°C or 26°C. The increase in primary root length over the following 5 d was measured and Glu sensitivity for each line is expressed as % inhibition compared to controls. The plots have been divided into four groups based on the pattern of temperature sensitivity (see text).

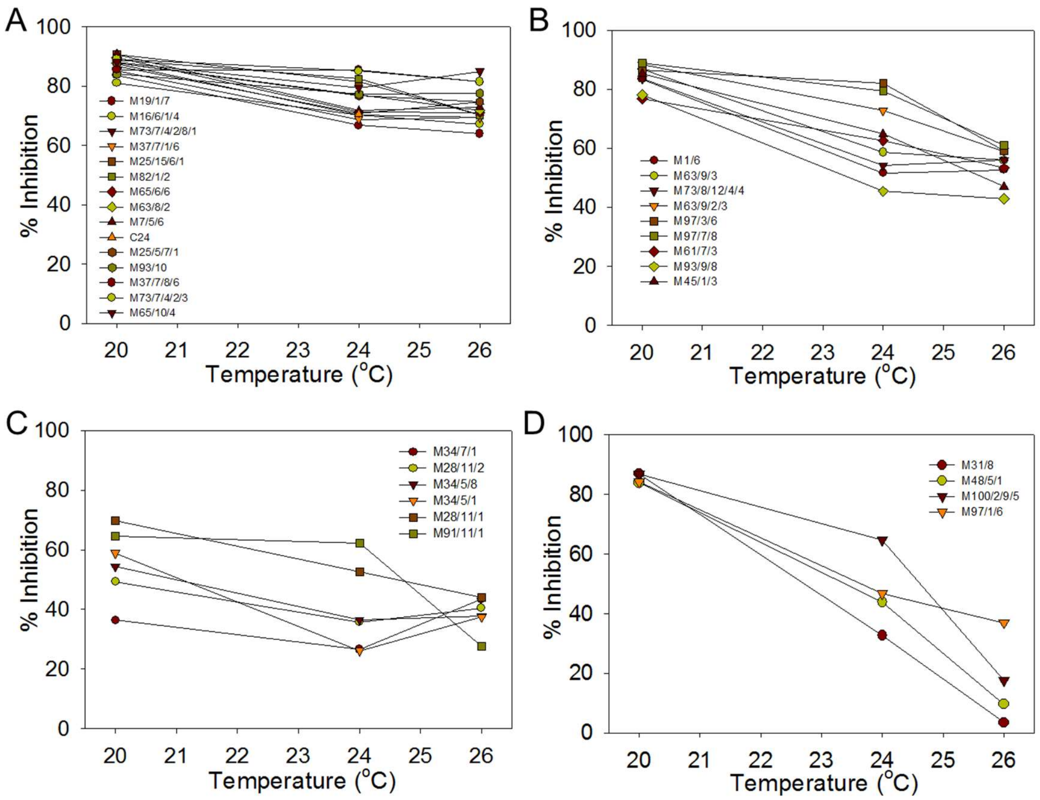

Fig. S8. Scatter plot showing the effect of nitrate on the glutamate sensitivity of a set of ILs in the C24 background. Each line was grown for 5 d on medium with or without 5 mM KNO_3 in the presence or absence of 50 μM glutamate and the percentage inhibition of primary root growth by glutamate was calculated relative to roots treated with the same nitrate concentration without glutamate (n = 6). The dotted line indicates the position on the graph where points would lie if nitrate completely failed to antagonize the glutamate effect. The data for C24 is shown with a red circle and four ILs that were analysed in more detail are indicated.

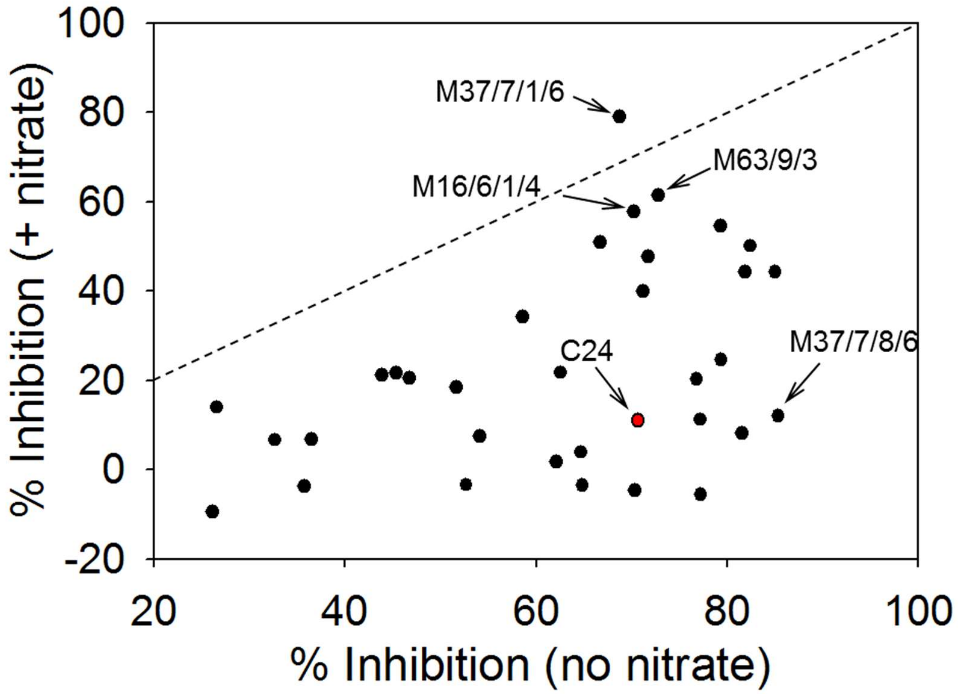

Supplement: supplementary_figures_S1_S8 [file erx132_suppl_supplementary_figures_s1_s8.pdf]
