# Supplementary material for: QTL analysis of the developmental response to L-glutamate in Arabidopsis roots and its genotype-by-environment interactions
Source: J Exp Bot. 2017 Apr 26;68(11):2919–31. doi: 10.1093/jxb/erx132 (PMC5853333; doi:10.1093/jxb/erx132)
Supplement: supplementary_tables_S1_S5 [file erx132_suppl_supplementary_tables_s1_s5.pdf]

QTL analysis of the developmental response to L-glutamate in Arabidopsis roots and its genotype-by-environment interactions

Pia Walch-Liu, Rhonda C. Meyer, Thomas Altmann and Brian G. Forde

SUPPLEMENTARY DATA

Table S1. Composition of basal medium (a diluted version of Gamborg's B5 medium).

Chemical	Final concentration
KCl	100 µM
$MgSO_4$	40 µM
$CaCl_2$	20 µM
NaH_2PO_4	22 µM
$MnSO_4$	0.9 µM
KI	90 nM
H_3BO_3	0.97 nM
$ZnSO_4$	0.14 nM
$CuSO_4$	2 nM
Na_2MoO_4	20.6 nM
$CoCl_2$	2.1 nM
Fe-EDTA	3.6 µM
2-[N-Morpholino]ethanesulfonic acid (MES)	2.6 mM
KOH	to adjust pH to 5.7
$CaCl_2$ + $MgCl_2$	1 mM each (to enable the Phytagel to set)

Table S2. CAPS and dCAPS primers used for fine mapping the *GluS1* locus

**Marker**	**Gene**	**Primers (5'-3')**	**Enzyme**	**Col-0 fragments (bp)**	**C24 fragments (bp)**	**SNP location on Chr 3^{a} (Col-0/C24)**
CAPS-1	At3g48090	F-TCTAGCTGTAACCCTAAGTTAAGACCA<br>R-GAGGTTTTTAAAGAAACTAGCATGG	*Mse*I	11, 18, 117, 142, 159	11, 18, 142, 276	17756110 (T/A)
dCAPS-1	At3g44830	F-ATTTTTTAGACCCGGTCAGATACT<br>R-TCCTAAAACCATCCATCATTACTT	*Spe*I	237	212	16369344 (G/A)
dCAPS-2	At3g45190	F-AAAGGGTTCTTGCTTTCTGTACTCA<br>R-ACAAAGGGCTCCAAAAACCAAT	*Dde*I	228	203	16542478 (A/G)
dCAPS-3	At3g46190	F-GAGCCGGTAAAGTTTGATAGTCTA<br>R-CCAAGTGAAAACAGGGTTGC	*Xba*I	209	185	16966518 (C/G)
dCAPS-4	At3g46880	F-TCAATGATGTATACTTGACTGTCT<br>R-CTATTGTTCCTCTTGTTGTACTA	*Spe*I	177	200	17266472 (C/T)
dCAPS-5	At3g47180	F-CCAAGAAATCATCCTCCTCCTCA<br>R-GCATGATTCGTCTCCCTCAAAG	*Hind*III	168	191	17373302 (G/T)
dCAPS-6	At3g48980	F-ACTCCTAATGTTACCCTCTCACC<br>R-CTCAGTGGAGTTCTGAGGGATCCC	*Sma*I	185	161	18157976 (T/C)

[a]The SNP locations are based on the TAIR10 annotation

Table S3. Summary of the QTL controlling the PBT trait in the Col-0/C24 RIL population based on CIM mapping of primary root length before transfer to glutamate

Environment		Exp't	QTL	peak	SI	Left marker	LOD	Allelic effect	pR^2	R^2 (%)	SD
Temp.	other										
21ºC	Low light	Col-0x C24	*PBT1*	1/22	18-24	MASC03658	6.94	-0.723	11.02	45.8	5.3
		C24x Col-0	*PBT1*	1/16	12-20	MASC09203	3.40	-0.466	6.07	6.10	3.5
24ºC	High light	1	*PBT2*	4/12	8-18	MASC04685	4.06	-0.864	25.56	33.2	8.2
		2	*PBT2*	4/10	8-16	MASC04685	5.19	-0.766	42.60	49.6	7.6
24ºC	High light +nitrate	1	*PBT1*	1/20	18-22	MASC03658	8.64	-1.016	23.74	30.6	8.2
			PBT3	5/24	20-28	MASC09209	5.09	-0.693	12.32		
		2	*PBT1*	1/20	18-24	MASC03658	4.65	-0.800	23.08	49.1	7.6
			PBT3	5/26	20-32	MASC09209	4.48	-0.510	19.73		
24ºC	shade	1	*PBT4*	4/48	44-52	MASC02548	4.32	-0.777	27.28	55.9	7.0
		2	*PBT4*	4/52	48-56	MASC02548	6.31	-0.966	29.71	39.7	8.1

Table S4. Map locations of introgressions in the ILs used in the present study based on mapping of framework P markers (Torjek et al., 2008).

C'some	Line (C24 background)	Introgressions from Col-0 (framework sites as defined in Table S3)	Notes	Line (Col-0 background)	Introgressions from C24 (framework sites as defined in Table S3)	Notes
**1**	**M82/1/2**	I.01		**N32/1/2**	I.01-I.03	
	**M100/2/9/5**	I.01-I.04		**N29/6/3**	I.0-I.05	
	**M91/11/1**	I.02-I.04/III.58-62	also carries introgression on Chr 3	**N87/2/7/8**	I.04-I.06	
	**M65/6/6**	I.03-I.09/II.44-II.46	also carries introgression on Chr 2	**N2/11/6**	I.05-I.12	
	**M34/5/1**	I.06-I.11/III.64-III.68	also carries introgression on Chr 3	**N42/2/7**	I.21-I.22	
	**M16/6/4/4/3***	I.18-I.23	not used in large-scale screen			
	**M37/7/1/6**	I.20-I.23				
	**M37/7/8/6**	I.22-I.23				
	**M16/6/1/4**	I.22-I.23				
**2**	**M73/8/12/4/4**	II.27-II.34		**N28/11**	II.41-II.46	
	**M1/6**	II.27-II.35		**N82/5/16**	II.44-II.46/IV.85-87	also carries introgression on Chr 4
	**M73/7/4/2/8/1**	II.40-II.43				
	**M73/7/4/2/3**	II.40-II.46				
	**M65/10/4**	II.44-II.46				
**3**	**M19/1/7**	III.47		**N88/1/2**	III.47	
	**M45/1/3**	III.47-III.56		**N88/2/9/2**	III.47/IV.74	also carries introgression on Chr 4
	**M63/8/2**	III.57-III.61		**N94/7**	III.47-III.50	
	**M28/11/1**	III.58-III.64		**N52/2**	III.53-III.63	
	**M28/11/2**	III.58-III.64/V.101-V.105	also carries introgression on Chr 5	**N81/4**	III.55-III.60	
	**M34/5/8**	III.64-III.68		**N21/3/14**	III.58-III.63	
	**M34/7/1**	III.64-III.68		**N28/13/8**	III.66-III.69	
	**M61/7/3**	III.64-III.69		**N28/6/1**	III.69	
	**M93/10**	III.65-III.66				
	**M93/9/8**	III.66-III.67				
**4**	**M63/9/2/3**	IV.70		**N38/3/1**	IV.81-IV.83	
	**M63/9/3**	IV.70-IV.74		**N62/3**	IV.81-IV.85	
	**M25/15/6/1**	IV.74-IV.84				
	**M25/5/7/1**	IV.78-IV.80				
	**M97/7/8**	IV.83-IV.87				
**5**	**M97/3/6**	V.93-V.96		**N58/2/12/5**	V.88-V.90	
	**M97/1/6**	IV.96/V.109-V.112	two separate introgressions on Chr 5	**N58/2/8/4**	V.88-V.91	
	**M31/8**	V.97-V.112		**N10/1/4**	V.90	
	**M48/5/1**	V.107-V.110		**N84/1**	V.90-V.94	
	**M7/5/6**	V.111-V.112		**N92/3**	V.90-V.94	
				**N21/3/11**	V.92-V.96	
				**N2/13/4**	V.95-V.104	
				**N22/8/2**	V.104-V.106	
				**N1/2/8**	V.104-V.106	
				**N95/2/6**	V.105-V.109	
				**N95/2/5**	V.105-V.111	
				**N70/10**	V.112	

Table S5. The framework SNP markers previously used to map the Col/C24 RILs and ILs (Torjek et al., 2003; Torjeck et al., 2006), their physical and genetic positions and the most closely associated gene.

Framework site	Framework marker	Physical position (nucleotides; TAIR10.0 release)	Genetical position (cM; JoinMap 3.0)	Corresponding or adjacent annotated gene
I.01	MASC03771	174605	0.00	At1g01471
I.02	MASC07014	1189375	4.51	At1g04410
I.03	MASC03758	2312698	8.79	At1g07520
I.04	MASC09203	3487274	12.42	At1g10560
I.05	MASC02475	4759728	16.07	At1g13920
I.06	MASC03658	5855333	19.83	At1g17130
I.07	MASC03911	7144336	22.23	At1g20620
I.08	MASC05029	8168391	26.47	At1g23050
I.09	MASC05303	9362052	30.28	At1g26970
I.10	MASC09223	10886897	36.63	At1g30680
I.11	MASC02998	11433410	39.01	At1g31850
I.12	MASC09204	12524970	42.71	At1g34320
I.13	MASC02577	13832436	48.07	At1g36700
I.14	MASC04127	15928392	49.93	At1g42400
I.15	MASC04209	16644717	51.93	At1g43886
I.16	MASC03340	17809681	57.26	At1g48240
I.17	MASC00545	18943965	61.26	At1g51140
I.18	MASC03754	20027196	64.97	At1g53645
I.19	MASC09205	20859471	67.37	At1g55805
I.20	MASC04170	22286231	71.81	At1g60490
I.21	MASC03447	24037255	76.30	At1g64680
I.22	MASC03631	25235673	81.66	At1g67350
I.23	MASC03684	26446765	88.42	At1g70220
I.24	MASC03930	27847120	93.15	At1g74045
I.25	MASC03765	28884867	96.38	At1g76900
I.26	MASC09206	30348203	101.04	At1g80745
II.27	MASC05502	133624	0.0000	At2g01250
II.28	MASC05477	1125048	7.4820	At2g03700
II.29	MASC06808	2026930	10.3220	At2g05520
II.32	MASC05857	4256265	13.8970	At2g10820
II.33	MASC05360	5179217	13.4850	At2g12660
II.35	MASC05657	7520366	24.6900	At2g17295
II.36	MASC02747	8429064	29.9020	At2g19450
II.37	MASC02600	9383832	33.3760	At2g22070
II.38	MASC02644	10428938	39.3070	At2g24550

II.39	MASC09221	11424765	42.5320	At2g26790
II.40	MASC06104	12519577	48.4820	At2g29125
II.41	MASC05434	13289706	52.3290	At2g31190
II.42	MASC09222	14375406	55.3230	At2g34030
II.43	MASC05386	15785611	60.7340	At2g37640
II.44	MASC06025	16886677	64.4080	At2g40435
II.45	MASC00371	18436623	69.2270	At2g44710
II.46	MASC02812	19671976	72.3940	At2g48100
III.47	MASC03898	276917	0	At3g01770
III.48	MASC03001	971403	2.01	At3g03800
III.49	MASC05312	2087109	5.78	At3g06652
III.50	MASC03344	3463230	10.75	At3g11040
III.51	MASC02947	4625764	13.98	At3g13990
III.52	MASC04608	5551390	17.71	At3g16350
III.53	MASC04279	6536028	20.2	At3g18950
III.54	MASC02841	7829504	24.37	At3g22180
III.55	MASC04516	8980164	32.25	At3g24620
III.56	MASC04523	9943490	38.02	At3g26950
III.57	MASC02648	11050930	45.99	At3g29070
III.58	MASC04262	12489387	49.48	At3g30805
III.60	MASC05045	14304561	50.04	At3g32300
III.61	MASC04819	15418717	52.25	At3g43530
III.63	MASC01171	16164006	57.09	At3g44590
III.64	MASC09224	18501466	67.69	At3g49900
III.65	MASC03218	19864963	73.95	At3g53580
III.66	MASC02788	20744711	78.11	At3g55920
III.67	MASC09218	21818753	80.91	At3g59030
III.68	MASC04925	22627912	82.33	At3g61140
III.69	MASC09219	23406914	85.53	At3g63380
IV.70c	MASC04123	301331	0	At4g00730
IV.71	MASC04725	1092491	5.88	At4g02485
IV.73	MASC09225	2907158	8.33	At4g05590
IV.74	MASC04685	5230768	9.56	At4g08280
IV.75	MASC02668	6120756	20.55	At4g09680
IV.76	MASC09213	7604892	33.51	At4g13020
IV.77	MASC03275	8961980	40.38	At4g15733
IV.78	MASC03263	9753853	43.08	At4g17490
IV.79a	MASC02548	10613944	46.87	At4g19470
IV.80	MASC04642	11786400	52.6	At4g22290

IV.81	MASC09214	12710203	55.99	At4g24620
IV.82	MASC09215	13588024	58.06	At4g27070
IV.83	MASC03154	14658631	60.4	At4g29950
IV.84a	MASC04005	15786556	63.08	At4g32717
IV.85	MASC04199	16879416	65.98	At4g35560
IV.86	MASC09216	17772130	70.83	At4g37800
IV.87	MASC05258	18538530	74.07	At4g39970
V.88a	MASC00144	506803	0	At5g02370
V.89	MASC04860	1193462	2.35	At5g04280
V.90	MASC04531	2073102	5.61	At5g06710
V.91	MASC09207	3284123	8.8	At5g10450
V.92	MASC05127	4243042	11.64	At5g13260
V.93	MASC03612	5209977	14.52	At5g15960
V.94	MASC09208	6539148	18.61	At5g19390
V.95	MASC09209	7717922	23.45	At5g23020
V.96	MASC03559	8810481	30.04	At5g25370
V.97	MASC04983	10095967	37.6	At5g28085
V.99a	MASC01174	12077175	40.8	At5g32440
V.100	MASC01361	13832746	42.97	At5g35630
V.101	MASC04275	14973938	48.75	At5g37690
V.102	MASC01582	16069652	53.87	At5g40190
V.103	MASC03128	17168921	59.33	At5g42810
V.104	MASC04317	18266163	62.17	At5g45160
V.105	MASC04298	19145448	64.69	At5g47140
V.106	MASC02675	20182226	67.04	At5g49680
V.107	MASC04591	21413372	70.72	At5g52310
V.108	MASC04394	22386815	75.35	At5g55170
V.109	MASC01545	23400832	78.78	At5g57760
V.110	MASC04576	24357567	81.81	At5g60590
V.111	MASC09211	25579812	86.26	At5g63920
V.112	MASC04350	26869691	92.62	At5g67350

1. Torjek O, Berger D, Meyer RC, Mussig C, Schmid KJ, Sorensen TR, Weisshaar B, *et al.*: **Establishment of a high-efficiency SNP-based framework marker set for Arabidopsis**. *Plant J* 2003, **36**: 122-140.

2. Torjek O, Witucka-Wall H, Meyer RC, von Korff M, Kusterer B, Rautengarten C, Altmann T: **Segregation distortion in Arabidopsis C24/Col-0 and Col-0/C24 recombinant inbred line populations is due to reduced fertility caused by epistatic interaction of two loci**. *Theor Appl Genet* 2006, **113**: 1551-1561.
